# Identification of a cell subpopulation with different response to lipid nanoparticles and effect of protein corona on uptake and transfection

**DOI:** 10.1016/j.mtbio.2026.103340

**Published:** 2026-06-10

**Authors:** Heba A. Fayyaz, Rixt I. Noordhof, Mia Fitriana, Anna Salvati

**Affiliations:** a Department of Nanomedicine and Drug Targeting, Groningen Research Institute of Pharmacy, Faculty of Science and Engineering, University of Groningen, Antonius Deusinglaan 1, Groningen, 9713 AV, the Netherlands; b Department of Pharmaceutics, Faculty of Pharmacy, Alexandria University, 1 Khartoum Square, Azarita, P.O. Box 21521, Alexandria, Egypt; c Department of Pharmacy, Faculty Mathematics and Natural Sciences, Lambung Mangkurat University, Jl. Jend. A. Yani KM 36, Banjarbaru, South Kalimantan, 70714, Indonesia

## Abstract

Lipid nanoparticles (LNPs) have enabled the approval of the first RNA-based drugs. However, there are still many aspects of their cellular behavior that remain unclear. Here, we used LNPs of composition comparable to Onpattro and we analyzed their uptake, transfection efficiency and effects in thousands individual HeLa cells by flow cytometry. This allowed us to determine the variability in outcomes in individual cells within the cell population. We found that upon incubation with the LNPs, a sub-population of cells with comparable uptake but much lower transfection, as well as signs of cellular stress and toxicity could be distinguished. This sub-population was observed with both empty LNPs and LNPs carrying RNA. Comparable outcomes were observed with a different LNP formulation and in other cell types. Moreover, when increasing LNP concentration, uptake, endosomal leakiness and transfection first increased, but then they decreased, and the higher uptake levels were associated with loss of cell viability. Characterization of the dispersions formed at different LNP concentrations showed that the amount of proteins adsorbed on the LNPs in medium with serum decreased at higher LNP concentrations. Similarly, uptake and transfection varied greatly when increasing serum concentration towards more physiological conditions. Overall, these results indicate that the response to LNPs is highly heterogeneous, and some cells within the cell population are more sensitive to the LNPs. Furthermore, LNP uptake and transfection are strongly affected by the modifications LNPs encounter upon dispersion in biological environments.

## Graphical abstract

**Figure:**
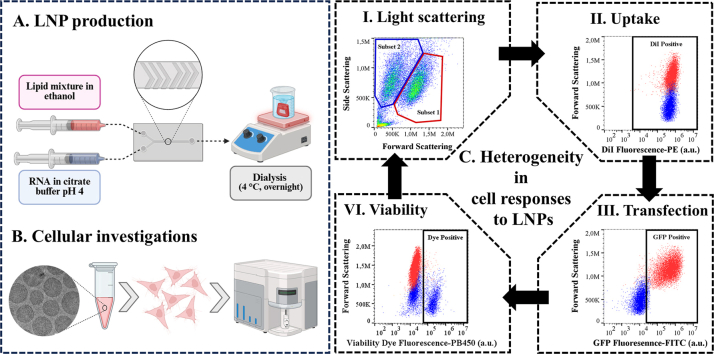

## 1 Introduction

Over the past 60 years, intensive research has been dedicated to the development of vectors for delivery of nucleic acids [1], starting from the sixties, a few years after RNA discovery, and until 2018, when the U.S. Food and Drug Administration (FDA) approved the first lipid nanoparticle-based RNA drug, Patisiran, (Onpattro, from Alnylam Pharmaceuticals) for the treatment of transthyretin amyloidosis [2,3]. Onpattro approval has revolutionized the field of therapeutic oligonucleotide delivery and thanks to the introduction of ionizable cationic lipids in their formulation, lipid nanoparticles (LNPs) have rapidly established as non-viral nano-vector not only for the delivery of siRNA, but also for mRNA, plasmid DNA, as well as therapeutic proteins [[3], [4], [5]]. The worldwide application of LNPs for mRNA delivery in the Biontech/Pfizer and Moderna vaccines against COVID19 in 2021-2022 further showcased the potential of this technology [3]. Since then, several clinical trials are currently taking place to investigate the potential of mRNA vaccines for different cancer types [6] and LNPs are being investigated also for *in-vivo* genome editing for treatment of cancer, genetic disorders, as well as infections [[7], [8], [9], [10], [11], [12], [13]].

Despite the tremendous potential of LNPs and extensive research on their use, there are still many aspects limiting LNP application, such as – among others – issues related to their stability, immunogenicity, poor intracellular delivery, as well as challenges in their application for extra-hepatic delivery [[14], [15], [16], [17]]. Additionally, mRNA-LNP transfection and protein expression efficiencies are known to vary considerably in different cells, and many cell types are still hard to transfect with this technology [18,19]. Many LNP parameters are known to be crucial determinants of their *in-vitro* and *in-vivo* efficacy, such as their lipid composition, size, shape, charge as well as apparent pk_a_ value [17,20]. In addition to LNP physico-chemical characteristics, multiple cellular and biological factors also affect and limit their efficacy. Among them, when looking at their behavior at the cell level, inefficient endosomal escape and poor intra-cellular delivery remain crucial aspects hindering their clinical translation [18,19,[21], [22], [23]]. In fact, several studies have showed that LNP endosomal escape efficiency is very minimal, with the majority of the particles remaining trapped inside the endo-lysosomal compartment, where they eventually are degraded [[21], [22], [23], [24]].

Cell to cell heterogeneity among individual cells within a cell population has also been observed. Several aspects are known to lead to cell-to-cell heterogeneity in nanoparticle uptake efficiency, such as variability in cell cycle, cell size and variability in gene expression among individual cells [[25], [26], [27], [28]]. However, in comparison, relatively less is known on the sources of variability in transfection in individual cells within a cell population, as well as – more in general – on how uptake correlates with transfection. Studies using LNPs for mRNA delivery reported that uptake is not a reliable predictor of intracellular delivery [24]. Similarly, more recently it was shown that differences in cell uptake do not directly correspond to differences in transfection [29].

Another aspect that is known to crucially affect nanoparticle behavior and interactions with cells, including for LNPs, is the modification that LNPs encounter upon contact with biological media [30,31]. Upon administration, the PEGylated lipids included in the LNP formulation have been shown to transfer from the LNPs into serum components [2,32,33]. Hence, as observed for most nanomaterials, LNPs adsorb a layer of biomolecules on their surface. Specific details in the composition of the resulting endogenous corona have been found to drive LNP distribution *in vivo* by endowing the LNPs endogenous targeting capability [[34], [35], [36], [37]]. For instance, Onpattro LNPs are known to adsorb a corona rich in apolipoprotein E which promotes targeting to the liver hepatocytes and interaction with the LDL receptor [2]. Similarly, it was shown that LNP transfection was different upon dispersion in lean and obese plasma, because of differences in the corona adsorbed on their surface [29,38]. More recently, it was shown that uptake and transfection followed a peculiar bell-shaped trend at increasing LNP content, as well as that they strongly varied when adding LNPs to cells in different serum concentrations [39]. Hence, it was suggested that an “optimal LNP-serum ratio” is required to enable efficient uptake and transfection [29,39]. While the protein to lipid ratio is known to affect corona composition, how this leads to differences in uptake and transfection remains to be elucidated.

Overall, despite their worldwide application, clearly there is still an urgent need of further studies to elucidate LNP-biological interactions at a fundamental level. This is required to understand how the modifications they encounter upon administration shape their interactions with cells, and also to understand their variable outcomes within individual cells, as well as among different cells within a population and in different cell types. Understanding better these aspects and the sources of these heterogeneities can help us improving LNP design in order to control their cellular behavior and enhance their efficacy.

Within this context, in this work LNPs with lipid composition similar to the clinically approved Onpattro particles were used and their interactions with HeLa cells were studied. HeLa cells are a common cell model for nanoparticle-cell studies, for which extensive information is already available, including on their interactions with LNPs. By taking advantage of flow cytometry, which allows to measure fluorescence, as well as side and forward light scattering of individual cells, we quantified uptake, transfection and cell response upon incubation with LNPs in thousands individual cells within the same cell population. This allowed us to determine cell-to-cell variability in LNP uptake and transfection among individual cells, as well as to identify sub-populations of cells with different response to the LNPs. Furthermore, by changing LNP concentration as well as serum amount, we determined how the modifications encountered in biological environments affect LNP uptake and transfection.

## 2 Methods

### 2.1 Lipid nanoparticle preparation

LNPs with composition similar to Onpattro were prepared via rapid mixing using a microfluidic set up [2]. The ionizable cationic lipid (6Z,9Z,28Z,31Z)-heptatriaconta-6,9,28,31-tetraen-19-yl 4-(dimethylamino)butanoate (D-Lin-MC3-DMA) (MedChem Express), the helper lipid 1,2-distearoyl-sn-glycero-3-phosphocholine (DSPC), the PEGylated lipid 1,2-dimyristoyl-rac-glycero-3-methoxypolyethylene glycol-2000 (DMG-PEG-2000), cholesterol (Avanti Polar Lipids), and the fluorescently labelled lipid 1,1′-dioctadecyl-3,3,3′,3′-tetramethylindocarbocyanine perchlorate (DiI, Sigma Aldrich) were dissolved in ultra-pure molecular biology grade ethanol (≥99.5% purity by volume, ThermoFisher Scientific) at 12.5 mM total concentration and 50:9.9:38.5:1.5:0.1 M ratio, respectively. The LNPs were loaded with either poly-adenylic acid (Poly-A 700-3500 KDa, Sigma Aldrich) as a common proxy for RNA, or reporter mRNA to produce green fluorescent protein (Cleancap eGFP mRNA 5moU, TriLink Biotechnologies). For all of the results presented (unless specified), the RNA was diluted in 10 mM citrate buffer (pH 4.0) to yield LNPs with 40:1 w/w total lipids: RNA ratio upon mixing with the organic phase (Note that this corresponds to a N/P ratio of ≈12 in case of eGFP mRNA cargo). The aqueous and organic solutions were mixed using a 125 μm etch depth hydrophilic micromixer chip (Dolomite; 3200401) at 1.2 mL/min total flow rate (TFR) and 3:1 volumetric flow rate ratio (FRR). Afterwards, particles were dialyzed against 1X PBS overnight at 4 °C using a Slide-A-Lyzer dialysis cassette (10K MWCO, ThermoFisher Scientific). For selected experiments, as specified in the corresponding figure captions, blank LNPs (without RNA) or LNPs with higher RNA content (10:1 w/w total lipid: RNA ratio) were prepared for comparison. Moreover, vortex mixing was used to produce LNPs of the same composition [40]. For this, the ethanol phase was pipetted into the aqueous phase at a 3/1 aqueous to ethanol ratio by volume to reach a final weight ratio of 40/1 (total lipids/RNA). The dispersion was, thereafter, mixed for 60 s using a Vortex-Genie 2 mixer at minimum speed. Next, particles were incubated for 10 min at room temperature before overnight dialysis at 4 °C against PBS. The recovered LNPs were quantified in terms of total lipid concentration, RNA entrapment efficiency, and total RNA content. For determination of total lipid concentration, a DiI-based fluorescence calibration curve was established by vortexing a lipid mixture of the same composition in ethanol into a solution of poly-A in 10 mM citrate buffer (pH 4.0). The dispersion was then mixed with a 2% triton X-100 (Sigma Aldrich) solution to disrupt the particles (1:2 v/v LNP dispersion to 2% triton X-100 ratio) and diluted serially in PBS to generate the fluorescence calibration curve. LNP samples were also treated with 2% triton X-100 in similar ratios and diluted in PBS to fit within the calibration curve linearity range. Samples were transferred into a black 96-well plate and their fluorescence measured (excitation-emission wavelengths 544-590 nm) using a Synergy H1 BioTek microplate reader. RNA entrapment efficiency was determined using the Quant-it RiboGreen RNA assay kit (ThermoFisher Scientific). Free un-entrapped RNA was quantified by the intensity of the fluorescence produced upon mixing the RiboGreen reagent with the intact LNP dispersion. Additionally, LNPs were subjected to lysis by treatment with 2% triton X-100 for extraction and quantification of total RNA content (both entrapped and un-entrapped). Both the intact and lysed LNP dispersions were diluted in 1X Tris-EDTA buffer and mixed in equal volumes with RiboGreen reagent. Fluorescence intensity (excitation-emission wavelengths 485-535 nm) was measured using a Synergy H1 BioTek microplate reader, and the percentage of entrapment efficiency was calculated as follows:

$$\%\;\text{Entrapment}\;\text{efficiency}=\frac{\text{Total}\;\text{RNA}-\text{Free}\;\text{RNA}}{\text{Total}\;\text{RNA}}\times 100$$

LNPs encapsulating poly A were stored at 4 °C for up to 30 days. LNPs with eGFP-mRNA were kept at 4 °C for maximum 2 days to be used freshly, otherwise they were stored at −80 °C after addition of sucrose to a final concentration of 8% w/v.

### 2.2 Lipid nanoparticle characterization

LNPs were characterized using dynamic light scattering (DLS) and zeta potential measurements. For comparison, DLS measurements were executed on two different commercial devices: a Malvern ZetaSizer Nano ZS (DLS instrument 1) and a Wyatt Technology Mobius (DLS instrument 2). In case of DLS instrument 1, results for each sample were recorded as an average of 3 measurements, each including 10 10-s runs at 20 °C. For DLS instrument 2, each sample was measured once as the mean value of 10 runs, 10 s each at 25 °C. Dispersions for DLS measurements were prepared at different total lipid concentration in water, PBS and cell culture medium supplemented with 10% Fetal Bovine Serum (both from Gibco ThermoFisher Scientific), hereafter referred to as cMEM. Particle size and concentration were also determined using nanoparticle tracking analysis (NTA) on a Malvern NanoSight NS300. Each measurement included 5 1-min videos, recorded at 20 °C. For this, particles were diluted in PBS to a concentration of 1 μg/mL total lipids. To test particle stability in biological conditions, LNP dispersions in cMEM incubated at 37 °C and 5% CO_2_ for 4 and 24 h were also characterized. For zeta potential measurements, a ZetaSizer Nano ZS was utilized. Results for each sample were recorded as an average of 3 measurements. The number of runs per measurement was set between 10 (minimum) and 100 (maximum), with analysis model specified as monomodal for high conductivity samples (LNP dispersion in PBS and cMEM).

LNPs were imaged using cryogenic transmission electron microscopy (Cryo-TEM). Particles (poly-A and mRNA loaded LNPs) were concentrated to a final total lipid concentration of 10 mg/mL by ultra-filtration using Nanosep devices with 10k Omega ultrafiltration membrane (Pall corporation, OD010C33) via centrifugation at 7000*g* for 10-15 min. 3 μL of LNP dispersion was added to glow-discharged carbon-coated grids (quantifoil 3.5/1, QUANTIFOIL Micro Tools GmbH) and rapidly-frozen using liquid ethane. Grids were examined with a Gatan model 626 cryostage in a Tecnai T20 Electron Microscope (FEI) operating at 200 keV. Images were acquired under low-dose conditions using a slow-scan CCD camera. Sample preparation and imaging were performed at the Cryogenic-Electron Microscopy Facility (Groningen Biomolecular Sciences and Biotechnology Institute, University of Groningen, The Netherlands). Samples were imaged at 62000X magnification.

### 2.3 Cell culture

Human cervical cancer epithelial cells (HeLa cells; ATCC CCL-2) were cultured in complete cell culture medium (cMEM) at 37 °C and 5% CO_2_. After thawing, cells were utilized for up to 18-20 passages. In addition, cells were checked regularly for mycoplasma to exclude contamination.

### 2.4 Uptake and transfection efficiency studies by flow cytometry

Uptake and transfection of LNPs encapsulating either poly-A or eGFP-mRNA in HeLa cells were evaluated by flow cytometry. Cells were seeded into 24-well plates (Garnier Bio-One) at a density of 50K and 30K cells/well for uptake studies up to 4 and 24 h, respectively. After 24 h at 37 °C and 5% CO_2_, LNPs were serially diluted in cMEM in the range 3 to 200 μg/mL of total lipid concentration (corresponding to roughly 0.05-5 μg/mL RNA for the 40:1 LNPs) and added to cells for either 4 or 24 h. For uptake kinetics studies, 30k cells/well were placed in 24-well plates for 24 h and then incubated with LNPs (12.5 μg/mL total lipid concentration corresponding to roughly 0.3 μg/mL RNA in cMEM) for different times.

To prepare samples for flow cytometry, after incubation with LNPs, cells were washed once with cMEM and twice with PBS to reduce the amount of adhering non-internalized particles. Thereafter, cells were harvested by incubation for 5 min with 0.05% trypsin-EDTA at 37 °C and transferred to flow cytometry tubes after mixing with cMEM to deactivate trypsin. Cells were spun down by centrifugation at 500*g* for 5 min and the cell pellet was resuspended in 100 μL PBS for measurements using a CytoFLEX S flow cytometer (from Beckman Coulter). The median fluorescence intensity (MFI) of DiI was recorded from the PE channel (excitation at 561 nm, emission at 585/42 nm) as a measurement of particle uptake, while the green MFI of eGFP was obtained from the FITC channel (excitation at 488 nm, emission at 525/40 nm) to determine transfection efficiency. FlowJo software (FlowJo_V10.8.1, LLC) was utilized for data analysis. For every experiment, two or three replicate samples were measured (as specified in figure caption) and their average and standard deviation calculated. Gates were set in FS-SS density plots to exclude debris and in FS by area against height to exclude doublets. Then, for each sample, at least 15k single cells were acquired for every sample (unless otherwise stated). For experiments with both DiI and eGFP signals, compensation was applied post-acquisition using FlowJo.

For LNP dose response experiments at different FBS concentrations, a series of poly-A LNP dispersions at different doses was prepared by diluting the particles in cell culture medium supplemented with a defined amount of FBS. Fixed volumes of FBS-containing MEM medium were added to fixed volumes of LNP dispersions at varying concentration in PBS. Next, cells were incubated with the LNPs for 4 h and then washed and harvested for flow cytometry as described above in order to determine uptake and transfection. Results were obtained from three replicate experiments, each with two replicate samples.

In order to test the effect of LNP pre-incubation with serum on cell uptake, LNPs were pre-incubated in 4 mg/mL and 40 mg/mL FBS at 37 °C and 5% CO_2_ at total lipid concentrations of 10 μg/mL and 100 μg/mL, for 4 and 24 h. After the pre-incubation, cells were treated with both pre-incubated and freshly made LNP dispersions for 2 h and then washed and harvested for flow cytometry as described above. The experiments were conducted three times, and for each condition two replicate samples were prepared.

### 2.5 Endosomal leakiness

To evaluate endosomal leakiness in response to different doses of LNPs, a calcein-based assay was employed [19]. Briefly, cells were incubated with varying doses of poly-A LNPs for either 4 or 24 h at 37 °C and 5% CO_2_. Following incubation, the LNP dispersion was discarded, and cells were subsequently incubated with 3 mM calcein (Merck Millipore) in cMEM for 15 min at 37 °C and 5% CO_2_. Next, cells were washed with PBS and incubated in cMEM for an additional 2–3 h before staining the nuclei with Hoechst (Sigma Aldrich, 1 μg/mL) and proceeding with live imaging. Confocal live-cell imaging was performed using Zeiss Celldiscoverer 7 microscope at 10X magnification.

### 2.6 Viability and cellular toxicity studies

HeLa cells were plated at a density of 30k or 50K cells/well in 24-well plates and incubated for 24 h at 37 °C and 5% CO_2_, prior incubation with increasing concentration of LNPs for 4 and 24 h, as described before. All experiments were conducted three times (n = 3), each with 2-3 replicate samples.

#### 2.6.1 MTT assay

Cell metabolic activity was quantified with a 3-(4,5-dimethylthiazol-2-yl)-2,5-diphenyltetrazolium bromide (MTT, Sigma Aldrich) assay. After incubation with the LNPs, cells were washed with 500 μL cMEM to remove particles adhering to cell membrane. Afterwards, each well was incubated with 250 μL of 0.5 mg/mL MTT solution in cMEM for 20-30 min at 37 °C and 5% CO_2_. Then, the MTT reagent was discarded, and dimethyl sulfoxide (DMSO) was added to each well (250-300 μL) to solubilize the formed formazan crystals. After 20 min shaking on a MedTec plate shaker (M − 1000) to ensure full solubilization of the formazan crystals, 200 μL from each well were transferred to a transparent 96-well plate. Absorbance was measured at 550 nm using a Synergy H1 BioTek microplate reader. The percentage of viable cells was determined by normalizing the results of cells incubated with the LNPs with those obtained from untreated control cells not exposed to particles, seeded and treated in the same way.

#### 2.6.2 Live/dead fixable viability dye eFluorTM 450 assay

In order to determine cell viability, the eBioscience Fixable Viability Dye eFluor™ 450 (ThermoFisher Scientific) was employed. It is an amine-reactive viability dye that can enter cells with compromised cell membrane and label them by irreversibly binding to intracellular proteins. Before the assay, the dye solution was defrosted and allowed to equilibrate to room temperature. A 2500-fold diluted dye working solution was prepared in DPBS (Gibco ThermoFisher Scientific). After the required incubation time with LNPs, cells were washed and harvested into flow cytometry tubes as previously described and stained with the dye working solution. In brief, after spinning down to pellet the cells and discard the deactivated TEP, cells were resuspended in 500 μL dye working solution and incubated for 30 min at 2-8 °C protected from light. Different dye incubation periods (30, 60 and 90 min) were tried to identify optimal conditions. Afterwards, 500 μL of freshly prepared 1% BSA (Sigma Aldrich) in DPBS were added onto cells suspended in the dye working solution. Cells were centrifuged at 500 *g* for 5 min, and the supernatant was discarded. Cells were washed once more with 500 μL 1% BSA in DPBS (FACS buffer) to remove any dye residues. Eventually, cells were suspended in 100 μL PBS for flow cytometry analysis. Results are plotted as the percentage of dye positive events after excluding both debris and doublets.

#### 2.6.3 Propidium iodide (PI)/FITC Annexin V assay

Dying cells with damaged cell membrane and apoptotic cells with exposed phosphatidylserine were identified by staining with propidium iodide (PI; Sigma Aldrich) and FITC Annexin V (BioLegend), respectively. In short, cells harvested in flow cytometry tubes after LNPs exposure were incubated with 100 μL of a mixture of PI (5 μg/mL) and Annexin V (200-fold dilution) dyes in 1X Annexin V binding buffer (BioLegend) for 15-30 min at room temperature protected from light. Next, 400 μL 1X Annexin V binding buffer was slowly added. Samples were placed on ice and mixed gently by pipetting up and down immediately before measurement by flow cytometry. For this assay, poly-A loaded LNPs without DiI were used to avoid interference with the viability dyes. For staining optimization, three different Annexin V dye diluted solutions (2000-fold, 200-fold and 20-fold) were tested on untreated control cells and cells treated with 0.5 μg/mL staurosporine for 5 h to induce apoptosis. Results are plotted as the percentage of double dye positive events. Based on staining optimization, a 200-fold dilution was used for further experiments.

### 2.7 Corona isolation and characterization

For corona studies, in order to form coronas equivalent to those formed on LNPs of 25, 50, 100, 150 and 200 μg/mL total lipids in cell culture medium with 10% FBS, samples at the same LNP to protein ratio were used [41]. To this end, 10 mg/mL poly-A LNPs were diluted to approximately 0.21, 0.42, 0.85, 1.27 and 1.7 mg/mL lipid in ∼77% FBS (roughly corresponding to 31 mg/mL total protein concentration) to produce 1 mL dispersions. The dispersion was incubated for 1 h at 37 °C and 300 rpm using a Thermo-Shaker (Grant Instruments Ltd). Thereafter, size exclusion chromatography (SEC) was performed to separate the corona-coated LNPs from excess free proteins using a Sepharose CL-4B column (15 × 1.5 cm; Sigma-Aldrich) pre-equilibrated with DPBS [42]. FBS at 31 mg/mL protein concentration was also run through the same column to exclude the presence of particles of same size and density (e.g. protein aggregates, exosomes or natural lipid nanoparticles) in the fractions in which LNPs were collected. In addition, LNPs at total lipid concentrations of 0.4 and 1.7 mg/mL in serum-free buffer and in FBS at 31 mg/mL protein were also run through SEC in order to compare DiI elution profiles and exclude transfer of DiI to serum components. Eluents were collected into 250 or 500 μL volume fractions and scanned for protein absorption at 280 nm and DiI lipid fluorescence (excitation/emission wavelengths 544/590 nm) using a Synergy H1 BioTek microplate reader. For selected conditions (as specified in text), the fractions with corona-coated LNPs were pooled together and their size distribution and zeta potential were determined as previously described. Next, samples were concentrated by centrifugation at 3000 *g* using an Amicon Ultra Centrifugal Filter, 100 kDa MWCO (Merck Millipore).

#### 2.7.1 Protein and lipid assays

After concentrating the pooled fractions of corona-coated LNPs, the amount of proteins adsorbed on the LNP surface was quantified using the Bio-Rad *DC* protein assay (Bio-Rad Laboratories, Inc.) following the manufacturer's instruction. 2% sodium dodecyl sulfate (SDS; Sigma Aldrich) was added to reagent B to avoid lipid interference. Calibration curves were obtained by serially diluting a BSA standard solution into several working solutions at known concentration. In brief, 5 μL of each standard and unknown sample solution was pipetted into a separate well in a transparent 96-well plate, combined with 25 μL of reagent A (supplemented with reagent S) and 200 μL of reagent B with 2% SDS. Samples were incubated for 15 min in dark conditions and absorbance at 750 nm was measured using a Synergy H1 BioTek microplate reader. The final lipid concentration was determined using DiI-based fluorescence calibration curves as described before. The protein/lipid ratio was calculated. The results obtained from at least 3 independent experiments are shown, together with their average and standard deviation.

#### 2.7.2 SDS-PAGE electrophoresis and silver staining

For selected LNP and FBS protein concentrations (as specified in text), the corona proteins recovered on the LNPs after isolation by SEC were separated by sodium dodecyl sulfate-polyacrylamide gel (SDS-PAGE) electrophoresis, as previously described [42,43]. Samples were loaded on a 4% stacking gel and 10% resolving gel after being mixed with 4X loading buffer (200 mm Tris-HCl, 400 mm DTT, 8% SDS, 0.4% bromophenol blue and 40% glycerol) and boiled at 95 °C for 5 min. Samples with similar protein amount (12-15 μg) were loaded in every lane in addition to FBS as a control and Precision Plus protein standards (Dual color, Bio-Rad Laboratories; 1610394). In order to compare the protein adsorbed in the different conditions, samples with similar lipid amounts were also loaded into the gels. Gels were run at room temperature first at 100 V till reaching the end of the stacking gel and then at 120 V when samples entered the resolving gel. Afterwards, gels were fixed for at least 1 h in a 50% (v/v) methanol, 10% (v/v) acetic acid solution, rinsed three times (30 min each) with 25% (v/v) ethanol and sensitized by soaking for 1 min in 0.02% sodium thiosulphate solution. Gels were washed with milli-Q water then impregnated in 0.2% silver nitrate solution for 20 min, washed again with milli-Q water then dipped into a color-rendering solution (4 × 10^−4^% sodium thiosulphate, 0.05% (v/v) formaldehyde and 6% sodium carbonate). When an adequate degree of staining was achieved, gels were washed with milli-Q water, and a stop solution was added (50% methanol and 10% acetic acid). Images were captured using UVITEC Alliance Q9 Advanced imager.

## 3 Results and discussion

### 3.1 LNP preparation and characterization

**Fig. 1:**
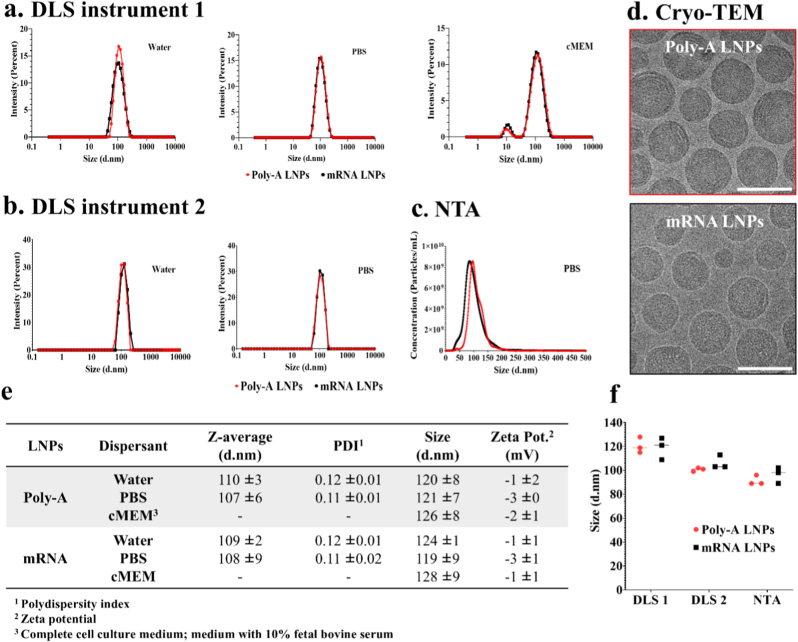
**Physico-chemical characterization of LNPs prepared using microfluidic mixing**. (a,b) Size distribution of 50 μg/mL LNPs in different media (water, PBS and cMEM) as measured by DLS. The results obtained with 2 different instruments are shown for comparison. (c) Size distribution of 1 μg/mL LNPs in PBS by NTA. (d) Cryo-TEM images of poly-A and mRNA loaded LNPs in 10 mg/mL total lipid concentration and 62000X magnification. Scale bar = 100 nm. (e) Physico-chemical characterization of poly-A and eGFP-mRNA LNPs (as obtained with DLS instrument 1). Measurements are the mean value ± standard deviation of 3 independent LNP batches. The Z-average and PDI obtained by cumulant analysis of the data are shown, together with the main peak size by distribution analysis (Size), as well as LNP zeta potential. (f) Hydrodynamic diameter by DLS (1 & 2) and by NTA. The values obtained for three independent batches of LNPs are shown together with their average, indicated by a line.

Onpattro-like neutral LNPs containing the ionizable cationic lipid D-Lin-MC3-DMA were prepared by ethanol dilution using a microfluidic mixer as described in the Methods. For all of the results presented (unless specified), particles were loaded with either poly-A or messenger RNA for the translation of the green fluorescent protein (GFP) at 40:1 w/w lipids to RNA ratio. Different total flow rates (TFRs) were tested for their effect on LNP colloidal properties, while maintaining similar flow rate ratio (FRR; 3:1 v/v aqueous to organic phase), as shown in Supp. Fig. 1. The obtained LNPs were characterized by dynamic light scattering (DLS), using two different instruments for comparison, as well as by nanoparticle tracking analysis (NTA). DLS measurements showed that smaller LNPs were obtained when increasing total flow rate (from 139 ± 4 nm to 77 ± 0 nm for dispersions in PBS when increasing the TFR from 0.6 to 6 mL/min, respectively), with comparable polydispersity index (PDI) and zeta potential. The same was observed by NTA, even though the decrease in particle size was not as pronounced as observed by DLS (from 98 ± 5 nm to 76 ± 1.5 nm, respectively, see Supp. Fig. 1e). Based on these results, a TFR of 1.2 mL/min was used to prepare the LNPs. Entrapment efficiency was high for both poly-A and mRNA particles (>90%, as determined by RiboGreen essay). Both poly-A and mRNA loaded LNPs had comparable particle size (∼120 nm and 105 nm by DLS and ∼95 nm by NTA, Fig. 1f), with a narrow size distribution and PDI values lower than 0.2 in water and PBS (Fig. 1a, b and e). Cryo-TEM imaging confirmed that the LNPs had stalked-like morphology (Fig. 1d), as expected. The LNPs formed stable dispersion also in medium supplemented with serum (cMEM), with a main peak at around 128 ± 9 nm, and a smaller peak at 10-30 nm from the free serum proteins in solution. Both LNPs retained a neutral zeta potential in either water, PBS or cMEM (Fig. 1e), consistent with their composition.

### 3.2 LNP uptake, transfection and endosomal leakiness in HeLa cells

**Fig. 2:**
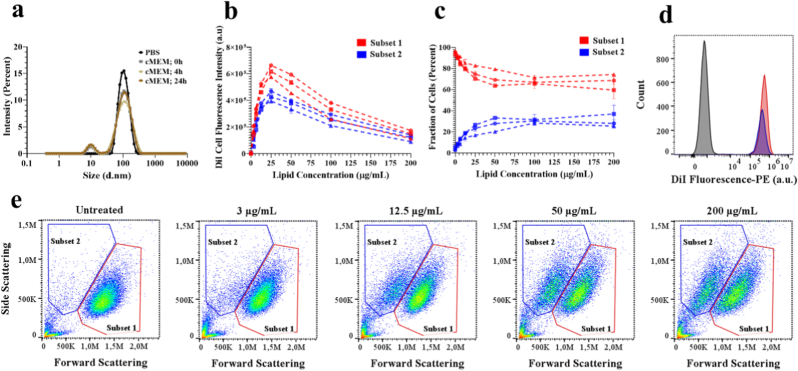
**Uptake of LNPs at different concentrations in HeLa cells for 4 h**. (a) DLS size distributions of LNP dispersions (50 μg/mL total lipids) in PBS and in cMEM after incubation for different times at 37 °C and 5% CO_2_. (b) Median fluorescence intensity values obtained by flow cytometry of cells treated with increasing concentrations of LNPs carrying poly-A for 4 h. Red and blue symbols show the results for cells belonging to sub-population 1 and 2, respectively, as defined in the gates shown in panel e. (c) Percentage of cells in each sub-population. (d) Fluorescence distributions of untreated cells (grey) and cells incubated with LNPs at 50 μg/mL total lipid concentration (subset 1 in red and subset 2 in blue). (e) Double scatter plots of forward versus side scattering of untreated HeLa cells as well as cells incubated with increasing concentrations of LNPs. The average and standard deviation of 2 replicate samples in 3 independent experiments are shown.

**Fig. 3:**
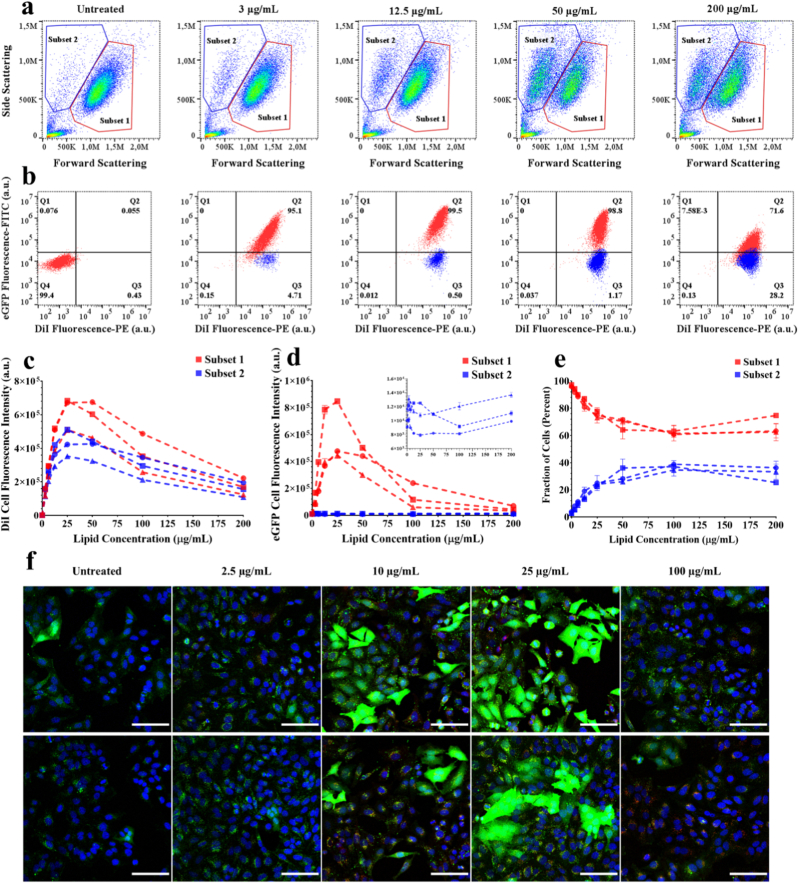
**Uptake, transfection and endosomal leakiness of HeLa cells incubated with LNPs**. (a) Forward – side scattering plots and (b) double scatter plots of transfection (eGFP fluorescence) versus uptake (DiI fluorescence) of untreated HeLa cells, as well as cells incubated with increasing concentrations of mRNA LNPs. (c-d) Median fluorescence intensity values of (c) uptake and (d) transfection in cells treated with increasing concentrations of mRNA LNPs for 4 h. Red and blue symbols show the results for cells belonging to sub-population 1 and 2, respectively, as defined in the gates shown in panel a. (e) Percentage of cells in each sub-population. The average and standard deviation of 2 replicate samples in 3 independent experiments are shown. (f) Confocal microscopy images of live HeLa cells incubated with increasing concentrations of poly-A LNPs (red) for 4 h followed by incubation with 3 mM calcein (green). Blue: Hoechst-stained nuclei. Scale bar: 100 μM. Two images from replicate samples are shown for each condition.

Human epithelial cervical cancer HeLa cells were used as a cell model commonly applied in the field to study LNP uptake and transfection efficiency (Fig. 2, Fig. 3) [21,44,45]. Characterization of the dispersions in cell culture medium with serum confirmed that the LNP dispersion remained stable also after 4 and 24 h incubation in the conditions used for the cellular studies (37 °C and 5% CO_2_) (Fig. 2a).

Dose response curves were generated by incubating cells for 4 h to increasing doses of poly-A LNPs (3-200 μg/mL in terms of total lipids, corresponding to roughly 0.05-5 μg/mL poly-A, as estimated based on the encapsulation efficiency and shown in Supp. Fig. 2a). Uptake efficiency followed a non-monotonic bell-shaped trend (Fig. 2b), as previously reported in primary human dermal fibroblasts and HuH-7 liver cells upon incubation with LNPs of comparable composition and at similar concentration ranges [39]. DLS measurements confirmed that stable dispersions with comparable size distributions were obtained at all LNP concentrations tested, hence excluding that the observed trend in uptake was due to loss of LNP stability (Supp. Fig. 2). Interestingly, instead, flow cytometry allowed to detect that incubation with LNPs led to the appearance of a second sub-population of cells with different light scattering properties. More specifically, as shown in Fig. 2e, upon incubation with the LNPs, next to the main population identified in the forward-side scattering plot for untreated cells (subset 1 cells, shown in red), a second sub-population could be distinguished (subset 2, blue), where cells had similar side scattering (SS), but smaller forward scattering (FS). Cellular debris and doublets were previously excluded from the analysis (see Supp. Fig. 3 for detailed gating strategy), hence confirming that the events detected in subset 2 were intact cells. The fraction of cells in this sub-population increased with increasing LNP dose up to around 40% (Fig. 2c).

Interestingly, the bell-shaped dose-response was observed for both the main population, as well as the 2nd sub-population (Fig. 2b and c). However, the fluorescence intensity of subset 2 cells was slightly lower than for cells in the main population (see overlapped cell fluorescence distributions in Fig. 2d), suggesting that for cells in this sub-population uptake efficiency was lower. The same two cell subpopulations could be distinguished upon incubation with LNPs produced by vortexing, which had a slightly larger particle size than those obtained by micro-mixing (∼150 nm compared to ∼120 nm respectively, Supp. Fig. 4). Comparable results were obtained for empty LNPs without RNA (Supp. Fig. 5), indicating that the appearance of this second sub-population of cells is induced by the LNPs themselves, regardless of their cargo. Having observed the appearance of a second cell sub-population upon incubation with the LNPs, further studies were performed in order to understand the factors leading to its formation and compare LNP behavior in this and the main cell population.

Shifts towards lower forward scattering in flow cytometry are often observed in case of cellular stress or toxicity [46]. Hence, we hypothesized that this second sub-population of cells could result from damage induced by the positive charges from excess tertiary amine groups of the ionizable lipids (not complexed with RNA) and that by increasing RNA load, the effect could be mitigated. To test this hypothesis, LNPs with higher RNA load were prepared (10:1 w/w lipid: poly-A ratio) (Supp. Fig. 6). However, also with these LNPs, 2 sub-populations of cells could be distinguished in the forward-side scattering plots, with a bell-shaped dose response in uptake.

In order to test whether the effect was limited to this particular LNP formulation and cell type, comparable experiments were performed with LNPs formulated with a different ionizable lipid (SM-102, as used for the Moderna vaccines against COVID19), as well as on different human cell types (more specifically in HuH-7 hepatocyte-derived carcinoma cells, HCT-15 colorectal carcinoma cells and A549 human adenocarcinoa alveolar basal epithelial cells). A second subpopulation of cells was detected also upon incubation with a different LNP (Supp. Fig. 7). Similar outcomes were obtained in the other cell types tested (Supp. Fig. 8). However, the amount of cells in the second subpopulation and the applied LNP dose and incubation time at which this subpopulation could be observed varied for the different cell types. This is expected given the variability in cell-dependent factors affecting cellular responses, such as for instance differences in uptake efficiency and intracellular processing, or in cell sensitivity and cell density, among others. Nevertheless, these results suggested that the appearance of a second subpopulation of cells in comparison to the main cell population was not limited to one specific LNP formulation and cell type.

We then used LNPs carrying the mRNA for the translation of the green fluorescent protein, GFP to determine how transfection efficiency varied for the two cell sub-populations. As shown in Fig. 3, after 4 h incubation, also for LNP carrying mRNA a second sub-population of cells with lower forward scattering was detected. The transfection efficacy mirrored the uptake trend, with a similar bell-shaped curve at increasing LNP concentration (Fig. 3b–d). A similar trend was observed also with a calcein-leakage assay by confocal microscopy on live cells [19]. For this assay, LNP encapsulating poly-A were used, instead of LNP carrying mRNA for GFP since GFP fluorescence would impair visualization of calcein. Following 4 h incubation, the number of cells with free dequenched calcein in the cytosol first increased and then decreased at higher LNP concentrations (Fig. 3f), suggesting differences in endosomal leakiness. No clear trend could be identified, instead, after 24 h incubation because of strong alterations of cell morphology and variability in replicate samples (Supp. Fig. 9). Overall, the observed trend in GFP fluorescence and calcein distribution is in line with what reported by Bates et al. for transfection efficiency and by quantification of number of galectin-9 puncta, respectively [39].

When comparing uptake in the 2 cell sub-populations, as observed with the LNPs carrying poly-A and empty LNPs, uptake was slightly lower for cells in subset 2 (Fig. 3b and c). Instead, when comparing transfection efficiency, for cells in subset 2 practically no GFP signal was detected after 4 h (Fig. 3c and d). After 24 h, transfection was detected also for cells in subset 2, and again a comparable bell-shaped trend could be observed, but overall with a much lower efficacy in comparison to the main cell population (Supp. Fig. 10). These results clearly indicated that the cells in the two subpopulations had comparable uptake properties, but the cells in subset 2 had strongly compromised transfection in comparison to the cells in subset 1. It would be interesting in the future to develop assays to be able to distinguish the two cell subsets also in relation to endosomal leakiness, in order to determine whether the loss of transfection observed for subset 2 cells is due to a lower endosomal escape capacity or alterations in the series of events following RNA release and leading to GFP translation.

We then used fluorescence double scatter plots to analyze in more detail how uptake (DiI) and transfection (eGFP) varied for individual cells within the cell population, as well as their correlation. The double scatter plots showed that uptake and transfection were correlated, hence cells with higher uptake also showed higher transfection. However, the distribution in cell transfection efficacy was much wider than for uptake, suggesting that the intercellular variability in transfection within a cell population is stronger than what observed for uptake. When comparing cells in the two subpopulations, double scatter plots clearly showed that the heterogeneity in uptake was comparable but that in transfection was much larger for subset 1 cells than in subset 2 cells. This is consistent with the observed drop of the overall transfection levels of subset 2 cells. Several factors such as cell size, cell cycle and variability in gene expression in individual cells are known to affect nanoparticle uptake efficiency [[25], [26], [27], [28]]. However, how these same factors affect transfection and whether additional ones determine variability in transfection is not known as of yet. The higher variability observed in transfection efficacy is consistent with the fact that after internalization, several other steps are required to achieve transfection and each of these steps likely contributes further to intercellular variability in response. Examples of additional factors affecting transfection include, for instance, differences in intracellular sorting of individual LNP after uptake, their capacity to destabilize the endosomal membrane and induce cargo release, as well as variability in factors affecting RNA translation and protein synthesis once the mRNA reaches the cytosol. At the same time, variability in LNP properties for individual particles within the formulation has already been reported and is likely to further contribute to the observed variability in LNP uptake and – especially - transfection. Indeed, a distribution in particle size and zeta potential is always observed (as we show here in Fig. 1). Furthermore, it is known that cargo encapsulation can also vary for individual particles, especially when loading larger RNA molecules into the LNPs, such as mRNA [47]. Despite the advances in methods for LNP preparation and the high quality of LNPs obtained by microfluidic mixing, empty LNPs without mRNA or LNPs carrying different amounts of RNA have been observed and this variability in individual LNPs within a sample likely contributes to the observed variability in their transfection.

Further studies to elucidate the sources of the observed variability in LNP transfection will help identify novel strategies to achieve better control in LNP intercellular and intracellular behavior, hence improving their efficacy.

### 3.3 Kinetics of LNP uptake and transfection

**Fig. 4:**
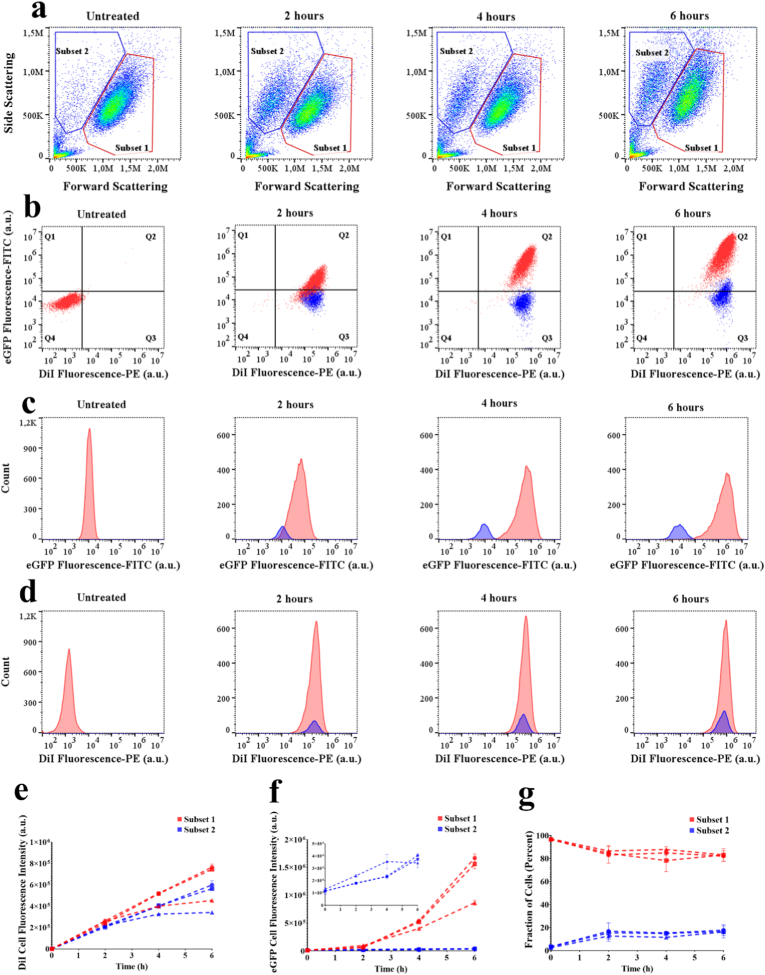
**Uptake and transfection kinetics of LNPs carrying the mRNA for GFP in HeLa cells**. (a) Forward – side scattering plots and (b) double scatter plots of transfection (eGFP fluorescence) versus uptake (DiI fluorescence) of cells incubated with LNPs at 12.5 μg/mL total lipids for increasing time. Red and blue symbols show the results for cells belonging to sub-population 1 and 2, respectively, as defined in the gates shown in panel a. (c-d) Fluorescence distributions of uptake (c) and transfection (d) at different incubation times (subset 1 in red and subset 2 in blue). (e-f) Median fluorescence intensity values of (e) uptake and (f) transfection as a function of time. (g) Percentage of cells in each sub-population. The average and standard deviation of 2 replicate samples in 3 independent experiments are shown.

As a next step, kinetics studies were performed in order to determine how early the second population of cells could be detected (Fig. 4 and Supp. Fig. 11). Uptake kinetics showed a linear increase followed by an apparent saturation, as usually observed in dividing cells [26]. Interestingly, even with a relatively low LNP concentration (12.5 μg/mL total lipids, corresponding to approximately 0.3 μg/mL RNA) the second sub-population of cells with lower forward scattering was visible already at short incubation time (2 h, see Fig. 4a and additional data in Supp. Figs. 11 and 12 for poly-A LNPs). The fraction of cells in this sub-population increased up to 20-40% of the total cell population (excluding debris and doublets) after 6 h to then decrease again to around 5-20% at 30 h (Supp. Fig. 11a and c). When looking at transfection efficiency, kinetics were comparable to what observed for uptake (Fig. 4 up to 6 h, and Supp. Fig. 13 for up to 30 h). As already observed upon incubation with different LNP doses, cells in the 2 sub-populations had comparable uptake, slightly lower in subset 2, while instead cells in subset 2 showed GFP signal only after 24 h (Supp. Figs. 13b,c,f). Cell fluorescence distributions and double scatter plots of transfection versus uptake showed again that both uptake and transfection varied among individual cells within the cell population, with a much wider distribution for transfection than for uptake (Fig. 4c and d), confirming a higher intercellular variability in transfection.

### 3.4 Effect of LNPs on cell viability

Changes in forward-side scattering distributions have been previously associated to cellular stress and cell damage [46]. Hence, we investigated whether incubation with the LNPs affected cell viability and whether the lower forward scattering cells identified upon incubation with LNPs (subset 2) were a sub-population of stressed and/or dying cells more sensitive to the LNPs.

**Fig. 5:**
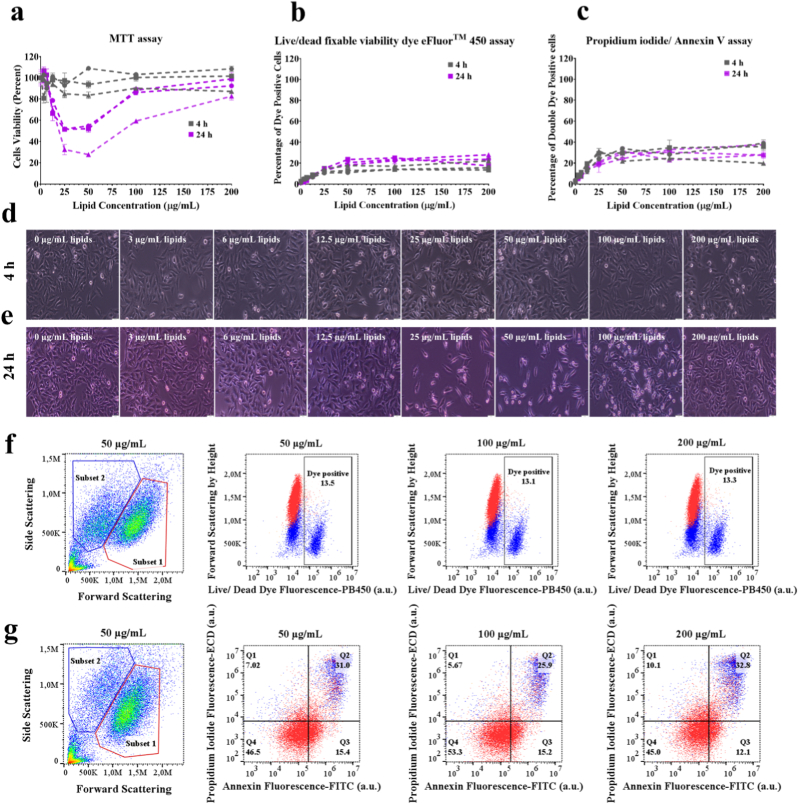
**Viability studies in HeLa cells**. Cell viability assessed by (a) MTT, (b) Live/dead fixable viability dye eFluor 450, and (c) Propidium iodide/FITC Annexin V staining after 4 and 24 h incubation with increasing concentrations of poly-A LNPs. The average and standard deviation of 2 replicate samples in 3 independent experiments are shown. (d-e) Light microscopy images of HeLa cells after incubation with increasing concentrations of LNPs for (d) 4 and (e) 24 h. (f-g) Forward – side scattering plots by flow cytometry of cells incubated with LNPs for 4 h and stained with (f) Live/dead fixable viability dye eFluor 450 and (g) propidium iodide/FITC Annexin V. In (f), double scatter plots of forward scattering versus eFluor 450 fluorescence are shown and in (g), double scatter plots of propidium iodide versus Annexin V fluorescence. Red and blue symbols show the results forcells belonging to sub-population 1 and 2, respectively, as defined in the gates shown in the forward – side scattering plots.

After 4 h incubation to increasing doses of LNPs, only minor effects on viability were observed in respect to untreated cells via a MTT assay (Fig. 5a). Instead, after 24 h, a decrease in viability was observed that mirrored the bell-shape trend in uptake, with up to 50-70% reduction of viability at the intermediate LNP doses for which higher uptake was observed (25-50 μg/mL total lipids and approximately 0.6-1.2 μg/mL poly-A). Consistent with these results, light microscopy images of cells incubated with LNPs at different concentrations showed no obvious differences after 4 h (Fig. 5d), while at 24 h, a decrease in cell density and dying cells were observed upon incubation with intermediate LNP doses (Fig. 5e). In the same conditions, a decrease in total cell count was observed by flow cytometry as well, with cell loss detected for both cell sub-populations (Supp. Fig. 14). Overall, these results suggest that the high uptake observed in cells incubated with intermediate LNP concentrations is associated with a decrease in cell viability. This is consistent with that reported by Bates et al., where it was also shown that the amount of endosomal escape, as measured by galectin 9 recruitment in endosomes, correlated with the amount of toxicity [39].

In order to be able to distinguish effects on viability for the 2 cell sub-populations, flow cytometry-based assays were used to detect eventual dead and dying cells with compromised cell membrane integrity. This was done using a fixable live/dead amine reactive dye (eFluor 450 dye), and with propidium iodide (PI)/Annexin V staining (Fig. 5f and g respectively; the corresponding gating strategy is included in Supp. Fig. 15). As shown in Fig. 5 b, a fraction of eFluor 450 dye positive cells was detected for cells incubated with 50 μg/mL total lipids (up to ∼20% cells), without further increasing at higher LNP concentrations. Interestingly, the eFlour 450 positive cells belonged to subset 2 cells, but not all subset 2 cells were labelled (Fig. 5f). The same was observed even when increasing the incubation time with the dye (Supp. Fig. 16). Instead, all subset 2 cells were stained by both PI and Annexin V (∼35-40%) (Fig. 5c, g and Supp. Fig. 17 for staining optimization). The different outcomes are likely due to differences in the capacity of each of the probes tested to diffuse inside cells when the cell membrane is damaged. Finally, both sub-populations of cells showed cell cycle distributions comparable to untreated cells (Supp. Fig. 18), hence excluding a correlation between subset 2 cells and the cell cycle.

Overall, these results indicated that the second sub-population of cells observed upon incubation with LNPs includes dying and/or dead cells with compromised cell membrane integrity. Bates et al. in their study showed that for cells incubated with increasing LNP concentrations, endosomal escape efficiency (as determined by the number of galectin 9 puncta) correlated with toxicity [39]. Based on these findings, one may hypothesize that the loss of viability detected in subset 2 cells may be due to a stronger endosomal escape efficiency in these cells. However, our results showed that subset 2 cells have comparable uptake in respect to the main cell population (slightly lower), but much lower transfection. In conclusion, our results indicated that upon incubation with LNPs and LNP uptake, cellular stress is induced in a sub-population of cells, resulting in alterations in their forward and side light scattering properties. These cells show comparable uptake behavior but with slightly lower efficiency, possibly because of the observed cell stress. Instead, in comparison, their transfection is drastically lower. This suggests that either these cells have a lower endosomal escape capacity or that the damage induced upon LNP uptake strongly compromises their translation machinery and indeed these cells include dying and/or dead cells with loss of cell membrane integrity. Hence, cells in this sub-population are cells more sensitive to the LNPs. Further studies are required to identify what are the causes leading to their different response to the LNPs. Identifying these factors could help optimizing LNP design in order to prevent similar cellular stress and damage, as well as to reduce the observed heterogeneity in LNP efficacy.

### 3.5 Effect of protein corona and serum content on LNP uptake and transfection

**Fig. 6:**
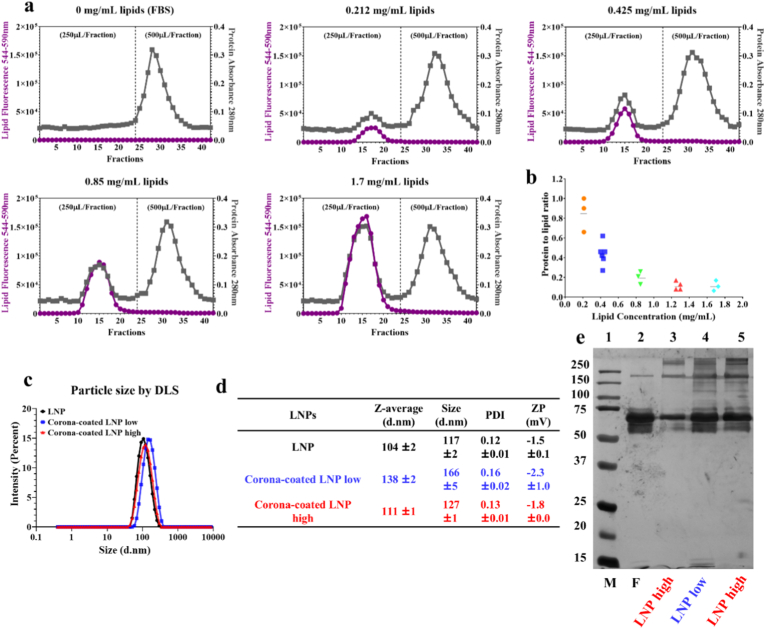
**Isolation and characterization of the corona-coated poly-A LNPs at different LNP concentrations**. (a) SEC elution profiles of FBS and LNP dispersions at different lipid concentrations. All samples included 77% FBS (roughly corresponding to ∼31 mg/mL total proteins, see Methods for details). (b) Amount of proteins adsorbed on LNPs after incubation with FBS: the protein to lipid ratio (μg of protein per μg lipid) obtained for 3-6 independent isolations is shown together with their average and standard deviation. (c) DLS size distributions of 50 μg/mL LNPs and the corona-coated LNP dispersions in PBS recovered by SEC. (d) Physico-chemical characterization of corona-coated LNPs (as obtained with DLS instrument 1). Results are the mean value ± standard deviation of 3 measurements. The Z-average and PDI obtained by cumulant analysis of the data are shown, together with the main peak size by distribution analysis (Size), as well as LNP zeta potential. (e) SDS-PAGE gel image of corona proteins recovered at different LNP concentrations. In each lane, the same lipid amount (lane 3 and 4), or protein amount (lane 4 and 5), was loaded. F: full FBS.

As a final step, further studies were performed to understand the observed trend in the LNP dose response. One aspect that is known to change when varying nanoparticle concentration is the corona adsorbing on the nanoparticle surface upon dispersion in serum [41]. More specifically, even when mixing the same nanoparticles and serum, changing the ratio between the nanoparticle amount (total surface area) and protein concentration leads to changes in the amount and/or identity of adsorbed proteins [41]. Previous studies where the bell-shaped trend in dose response was first reported suggested that an ideal protein to lipid ratio exists, at which uptake and transfection efficiency are optimal [39]. In order to study this further and determine whether this trend is indeed connected to differences in the corona adsorbed on the LNPs, we used size exclusion chromatography (SEC) to isolate the corona-coated LNPs obtained after incubation at different LNP-to-protein ratios, mirroring the conditions used in the dose response studies (roughly corresponding to the ratio used when dispersing 25 to 200 μg/mL LNPs in cell culture medium with 10% FBS. See Methods for details) [42]. Fractions containing corona-coated LNPs were identified by measuring DiI fluorescence to detect the LNPs (Fig. 6a). Of note, the DiI fluorescence SEC elution profiles obtained for LNPs in a serum-free buffer and the same amount of LNPs after dispersion in FBS were identical and no DiI signal could be detected in the fractions in which free FBS proteins eluted. This indicated that DiI remains associated to the LNPs and does not transfer from the LNPs to serum components, confirming that DiI fluorescence can be used as a reliable readout for identifying and quantifying LNPs uptake by cells (Supp. Fig. 19). The SEC elution profile obtained for FBS alone (protein absorbance at 280 nm) confirmed that no endogenous serum components were detected in the fractions in which the LNP eluted (also in Fig. 6a). Instead, absorbance at 280 nm was detected also for LNPs in a serum-free buffer in the fractions in which LNPs eluted (Supp. Fig. 20). Because of this and in order to determine protein amounts with higher accuracy, a protein assay was used to determine the amount of corona proteins adsorbed on the isolated corona-coated LNPs and to calculate from this the protein to lipid ratio at the different LNP concentrations (see Methods for details).

Interestingly, the results showed that the amount of adsorbed proteins progressively decreased at increasing LNP concentrations (Fig. 6b), corresponding to the conditions where a lower uptake by cells was observed. Further studies were performed for the corona-coated LNPs formed at a low and a high LNP content (roughly corresponding to the ratio used when dispersing 50 and 150 μg/mL LNPs in cell culture medium with 10% FBS, here referred to “LNP low” and “LNP high”, respectively. See Methods for details). DLS showed that the isolated corona-coated LNPs had homogenous size distributions at higher size than the bare LNPs (166 ± 5 nm and 127 ± 1 nm, at the lower and higher LNP concentration, respectively) (Fig. 6c and d). The lower amount of adsorbed proteins could explain the smaller size observed for the corona-coated LNP at higher LNP content (Fig. 6c and d). Consistent with corona protein quantification, SDS-PAGE of the recovered corona-coated LNP confirmed that although the band pattern was similar for the two samples, a lower amount of proteins was adsorbed on the LNP at higher concentration (Fig. 6e). Further studies are required to compare the composition of the adsorbed proteins and identify specific corona components (or corona patterns) that may favor LNP uptake and transfection. Nevertheless, these results clearly showed that differences in uptake efficiency correlated with differences in the amount of proteins adsorbed on the LNPs in serum. Proteins favoring uptake may adsorb on the LNPs at intermediate concentrations for which a higher uptake was observed, while they may be depleted and/or displaced by other corona components when increasing LNP content further, hence leading to the observed decrease in uptake and bell shaped trend at higher LNP doses.

**Fig. 7:**
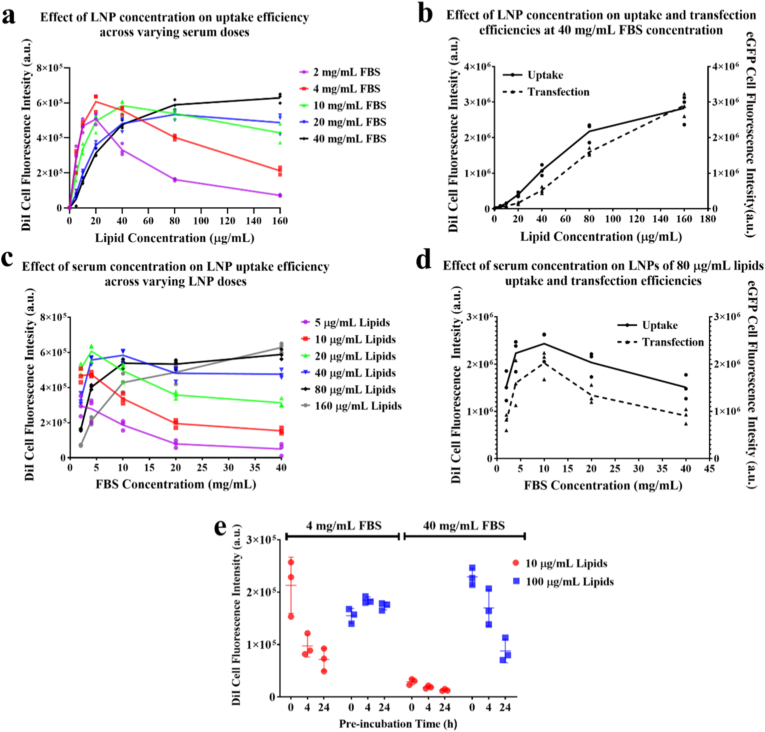
**Effect of serum concentration on LNP uptake and transfection**. Median fluorescence intensity values obtained by flow cytometry of cells treated for 4 h with increasing concentrations of (a and c) LNPs carrying poly-A and (b and d) LNPs carrying mRNA in different concentrations of FBS. The results of panel a are shown again in panel c as a function of FBS concentration for comparison. (e) Uptake of LNPs at two concentrations (10 and 100 μg/mL total lipids) after different pre-incubation time in serum-containing media (4 or 40 mg/mL FBS, roughly corresponding to 10% and full FBS) at 37 °C and 5% CO_2_. The results obtained in three independent experiments (each with 2 replicate samples) are shown together with their average, indicated by a line (In e, the standard deviation is also included).

Connected to this, Bates et al. showed that when varying serum content between 0 and 10% FBS, uptake and transfection kinetics could be modulated, likely due to similar effects on corona composition and amount of adsorbed proteins [39]. Here instead, given the observed effects of the protein to lipid ratio on the adsorbed corona, we further challenged the LNPs by incubation at increasing concentration of FBS up to full serum (with 100% FBS roughly corresponding to 40 mg/mL proteins), in order to simulate more physiological serum concentrations and quantify how uptake and transfection varied under these conditions. The results showed that increasing serum concentration strongly altered the LNP dose-response curve (Fig. 7a). More specifically, the described bell-shaped dose response was observed only at low FBS concentrations (corresponding to 10% FBS and lower, as also reported by Bates *et*. *al*. [39]). Instead, when increasing FBS content (above 10% FBS) to concentrations closer to physiological conditions, uptake increased linearly at increasing LNP doses, and transfection efficiency closely mirrored this trend (Fig. 7b). When looking at the effect of FBS concentration on uptake (Fig. 7c), at low LNP concentrations, uptake decreased at increasing FBS content. A lower uptake is usually observed when adding particles to media with increasing protein concentration. This is usually attributed to a higher competition for uptake from the increasing free proteins in solution [43,48]. Instead, at higher LNP concentration (>40 μg/mL total lipids), increasing FBS concentration led to an increase in LNP uptake (Fig. 7c). Similar results were observed also when measuring transfection for mRNA-LNPs at a 80 μg/mL total lipids (corresponding to ≈ 2 μg/mL mRNA) added to cells at increasing serum concentrations (Fig. 7d).

Finally, we tested whether the observed differences in uptake at different serum concentrations could also be connected to effects of serum content on PEG shedding. A previous study by Gallud. A*.* et al. showed that uptake was higher when LNPs were pre-incubated with serum for increasing time before exposure to cells [33]. This effect was attributed to progressive PEG shedding and the subsequent maturation of the protein corona, which together facilitate more efficient uptake by cells. As shown in Fig. 7e, instead, pre-incubating LNPs with 10% FBS or full FBS (roughly corresponding to 4 and 40 mg/mL proteins) resulted in most cases to a reduction in uptake. Hence, in the conditions tested, the observed differences in uptake at different serum concentration could not be directly attributed to effects on PEG shedding and corona maturation.

Collectively, these findings further iterate that the modifications LNPs encounter upon dispersion in biological media strongly modulate the following interactions with cells, affecting both their uptake and transfection efficacy [20,33,[38], [39], [40]].

## 4 Conclusion

In this work, we studied the uptake behavior and transfection of neutral LNPs encapsulating large RNA molecules in HeLa cells. By taking advantage of flow cytometry, we measured the fluorescence and light scattering properties of thousands individual cells. This allowed us to detect the development of a second cell subpopulation with distinct forward scattering in comparison to the main cell population. This sub-population of cells becomes visible even at low concentration and short exposure time and shows slightly reduced uptake, but strongly lowered transfection. The alteration in light scattering properties as well as the loss of cell membrane integrity and viability in these cells indicate that these are cells more sensitive to the LNPs, with strongly compromised translation machinery. The reasons behind the different response need to be further elucidated and differences in endosomal escape may in part contribute to it [39]. Identifying the factors leading to the different cellular response may help to design LNPs where similar effects are avoided, and overall with a lower variability in outcomes and efficacy.

In addition to this, by varying LNP and serum concentrations, we studied how uptake and transfection are affected by the modifications LNPs encounter upon dispersion in biological media. In agreement with recent findings in the literature, uptake, transfection, and endosomal leakiness followed a bell-shaped trend, with a decrease in cell viability at the intermediate LNP concentrations, where all of them were higher [39]. The bell-shaped trend was observed for cells in both of the identified cell sub-populations. Changing the lipid to protein ratio when varying the LNP or serum concentration is known to affect protein corona formation. In order to determine whether the observed trend was indeed connected to differences in the corona formed on the LNPs, we isolated the corona-coated LNP formed at different LNP concentrations in the same dose-range used for cell studies. This allowed us to determine that the amount of corona proteins adsorbed on the LNPs decreased at the higher LNP concentrations, for which a lower uptake and transfection were observed. This suggested that when increasing LNP content, corona proteins favoring uptake are depleted and/or may be displaced by other components that lower uptake efficiency, hence leading to the observed decrease in uptake, endosomal leakiness and transfection. Previous studies showed that uptake and transfection varied also when varying FBS content between 0 and 10% [39], suggesting the presence of an optimal protein to lipid ratio that favors LNP efficacy. To further challenge these observations, we determined uptake and transfection when increasing serum content towards more physiological concentrations, up to full FBS. Interestingly, in some conditions, contrary to what it is usually observed, we found that both uptake and transfection increased when increasing serum content. These findings re-iterate the strong impact of the modifications the LNPs encounter upon administration in biological fluids on their uptake and cell behavior [38,39]. Characterizing in detail the composition of the corona adsorbed on the LNPs in these conditions in order to identify the corona proteins promoting uptake may help to develop novel strategy to improve LNP uptake and transfection in physiological conditions.

## CRediT authorship contribution statement

**Heba A. Fayyaz:** Conceptualization, Formal analysis, Investigation, Methodology, Visualization, Writing – original draft. **Rixt I. Noordhof:** Investigation, Writing – review & editing. **Mia Fitriana:** Investigation, Writing – review & editing. **Anna Salvati:** Conceptualization, Methodology, Supervision, Writing – original draft.

## Declaration of competing interest

The authors declare that they have no known competing financial interests or personal relationships that could have appeared to influence the work reported in this paper.

## Data Availability

Data will be made available on request.
