# Supplementary material for: Identification of a cell subpopulation with different response to lipid nanoparticles and effect of protein corona on uptake and transfection
Source: Mater Today Bio. 2026 Jun 10;39:103340. doi: 10.1016/j.mtbio.2026.103340 (PMC13280413; doi:10.1016/j.mtbio.2026.103340)
Supplement: Multimedia component 1 [file mmc1.docx]

# Supplementary Information

# Identification of a cell subpopulation with different response to lipid nanoparticles and effect of protein corona on uptake and transfection

# Heba A. Fayyaz, Rixt I. Noordhof, Mia Fitriana, Anna Salvati

**Supplementary Methods**

## *Cell cycle distribution analysis*

In order to investigate potential variations in cell cycle distribution between the two cell subpopulations, subset 1 and 2, after incubation with DiI-free LNPs, cells were washed and harvested into flow cytometry tubes as described in the main Methods. Then, cells were treated with the live/dead fixable viability dye eFluor^TM^ 450 as explained in the main Methods to label cells belonging to subset 2. Afterwards, cells were incubated for 1 h in ice-cold 70% ethanol for fixation and cell membrane permeabilization. The supernatant was discarded carefully after centrifugation at 500 *g* for 5 min to pellet down cells. Cells were resuspended in PBS and spun down once more by centrifugation at 500 *g* for 5 min to wash and discard any residual ethanol. The washed pellet was resuspended in 50 µL RNase solution (1 mg/mL stock solution) and incubated for 4-5 min in room temperature to eliminate any possible interference from RNA molecules. Finally, in each tube, 12.5 µL of a 1 mg/mL PI solution was added, together with DPBS to reach a final volume of 500 µL. Samples were incubated for 30 min at room temperature and then measured by flow cytometry. Results were obtained from one independent experiment with 3 technical replicates.

*LNP absorbance spectrum*

The absorbance between 220 and 350 nm of empty LNPs (no-RNA) and LNPs loaded with poly-A (40:1 w/w total lipid to RNA ratio), with and without DiI, at a total lipid concentration of 1 mg/mL, was determined using a NanoDrop One (ThermoFisher Scientific). The A280 setup was configured using the general reference setting for protein quantification, where an absorbance of 1.0 at 280 nm corresponds to a 0.1% (1 mg/mL) protein solution.

*Preparation and characterization of SM-102 based lipid nanoparticles*

LNP with the same molar composition as Onpattro, but with the ionizable lipid SM-102 (heptadecan-9-yl 8-[2-hydroxyethyl-(6-oxo-6-undecoxyhexyl)amino]octanoate; MedChem Express) instead of D-Lin-MC3-DMA were prepared as described in the Methods, using the same microfluidic setup and mixing conditions. The LNP were labelled by incorporating DiI and were loaded with poly-A at 40:1 w/w total lipid to poly-A ratio. Particles characterization was performed by dynamic light scattering using a Malvern Zetasizer Nano ZS. Dispersions containing 50 µg/mL total lipids were prepared in water, PBS, and cMEM. For each sample three measurements were acquired, with each measurement consisting of ten 10-second runs at 20 °C. For evaluation of cellular responses, SM-102 LNPs were serially diluted in cMEM to final total lipid concentrations ranging from 3 to 200 µg/mL, corresponding to approximately 0.05–5 µg/mL poly-A. LNPs were incubated with HeLa cells for 4 h, washed, harvested and subjected to flow cytometry analysis as described in the Methods.

*Uptake studies in various cell lines*

In order to quantify LNP uptake in different cell types, additional uptake studies were performed with HuH-7 hepatocyte-derived carcinoma cells, HCT-15 colorectal carcinoma cells and A549 human adenocarcinoma alveolar basal epithelial cells. Cells were seeded in 24-well plates at densities of 40k, 100k, and 50k cells/well, respectively. LNPs were serially diluted in cell culture medium with 10% FBS to achieve total lipid concentrations ranging from 3 to 200 µg/mL, corresponding to approximately 0.05-5 µg/mL RNA. After 4 h or 24 h incubation, cells were washed and harvested for flow cytometry analysis as described in the Methods.


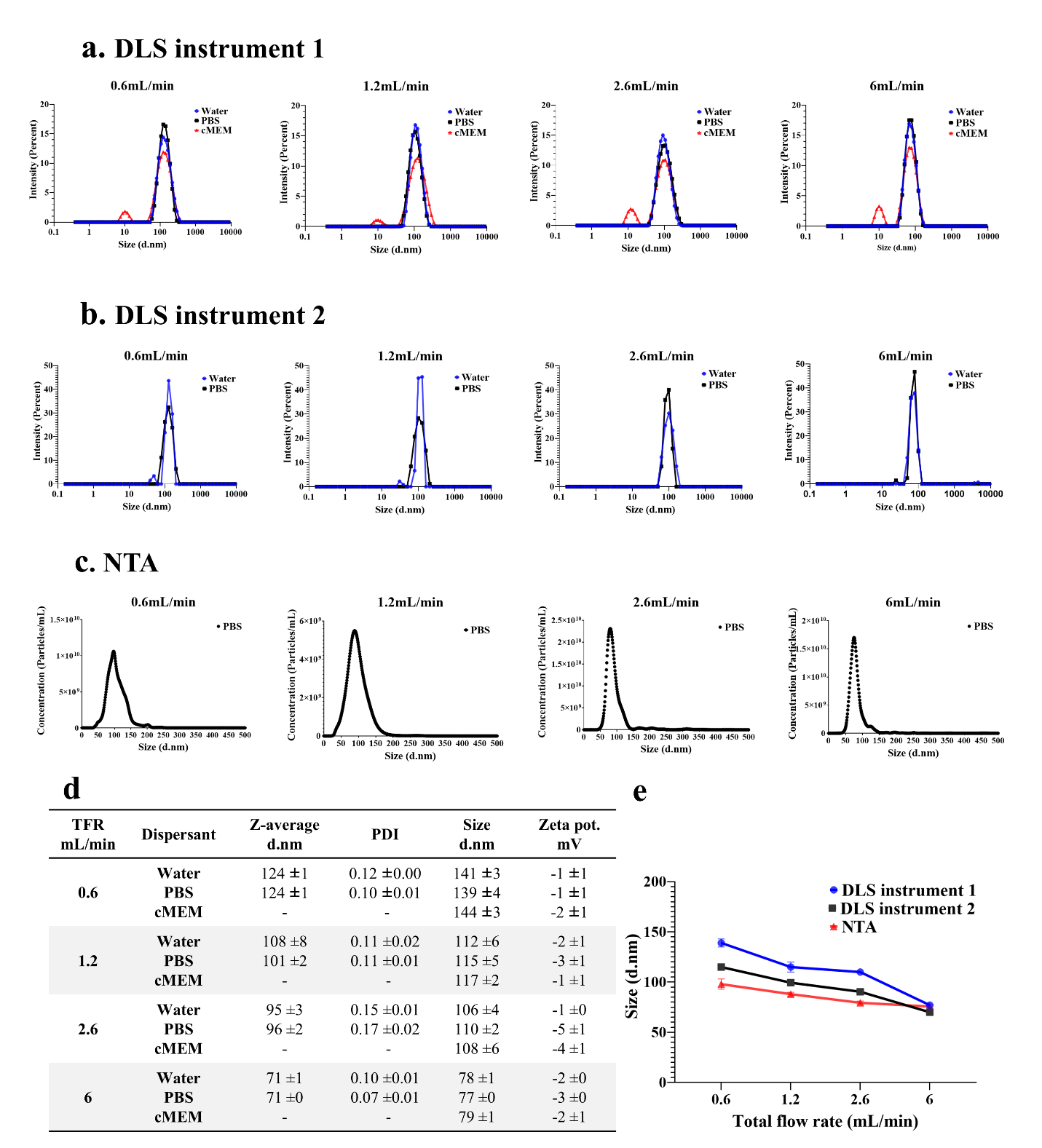


Supplementary Fig. 1. Physico-chemical characterization of poly-A LNPs with 40:1 w/w lipid to poly-A ratio as a function of total flow rate (TFR) at constant flow rate ratio (FRR) of 3:1 v/v aqueous: ethanolic phase. (a, b) Size distribution of 50 µg/mL LNPs (total lipids) in different media (water, PBS and cMEM) as measured by DLS. The results obtained with 2 different instruments are shown for comparison. (c) Size distributions of 1 µg/mL LNPs in PBS by NTA. (d) Physicochemical characterization of poly-A LNPs as measured by DLS instrument 1. The Z-average and PDI (polydispersity index) obtained by cumulant analysis of the data are shown, together with the main peak size by distribution analysis (Size), as well as LNP zeta potential. The results are the mean and standard deviation of 3 measurements of the same LNP batch. (e) Hydrodynamic diameter by DLS (1 & 2) and NTA. Values are displayed as an average of 3 measurements for DLS 1, 10 runs in case of DLS 2, and 5 different videos for NTA.


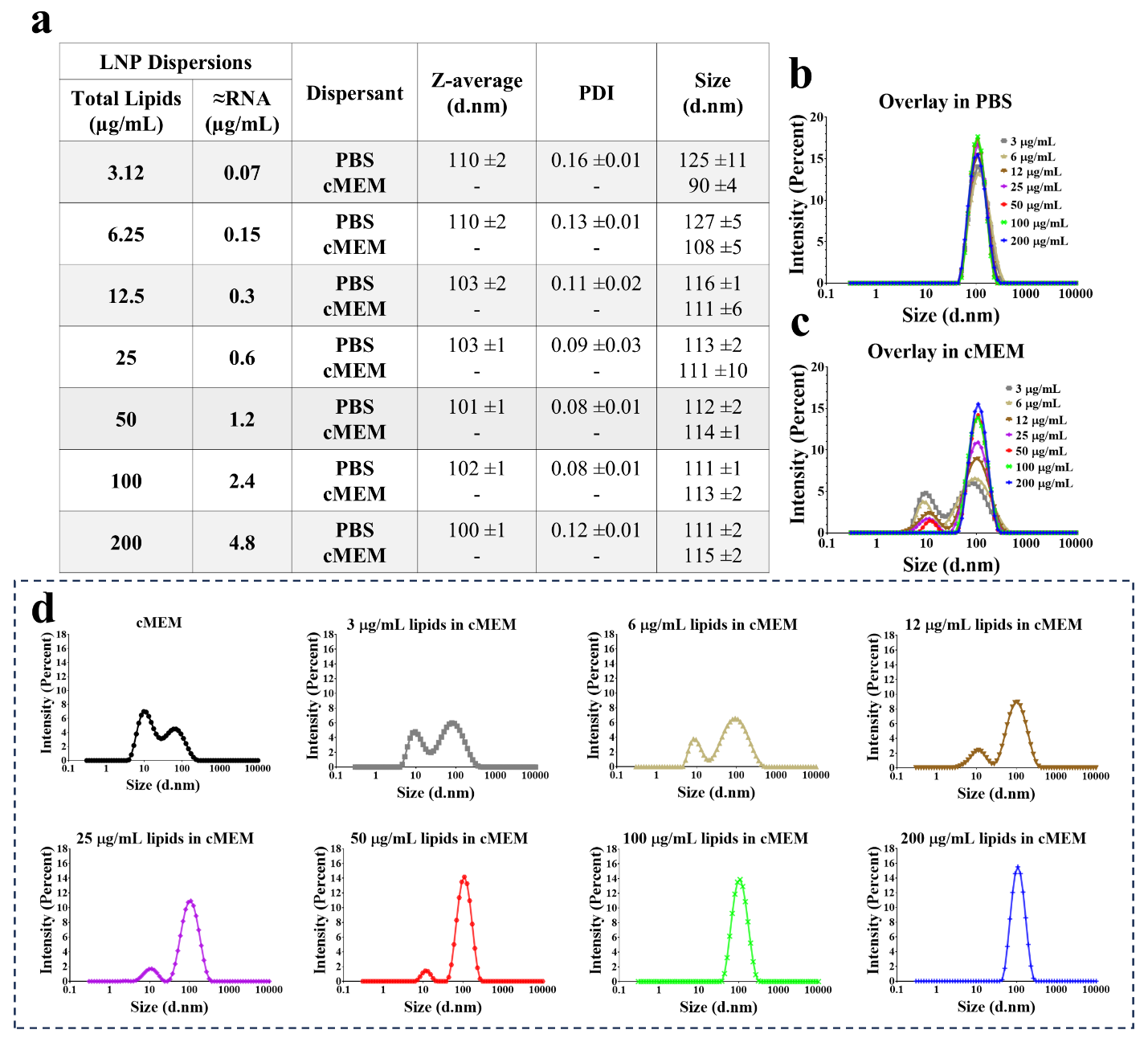


**Supplementary Fig. 2. Physico-chemical characterization of poly-A LNPs (40:1 w/w) at different concentrations.** (a) Physicochemical characterization of poly-A LNPs as measured by DLS. The Z-average and PDI obtained by cumulant analysis of the data are shown, together with the main peak size by distribution analysis (Size). The results are the mean and standard deviation of 3 measurements of the same LNP batch. (b, c) Overlay of size distributions of poly-A LNPs at varying concentrations in PBS and cMEM, as measured by DLS. (d) Size distribution profiles of cMEM alone and of poly-A LNPs at different concentrations dispersed in cMEM. Stable dispersion could be obtained at all tested concentrations.


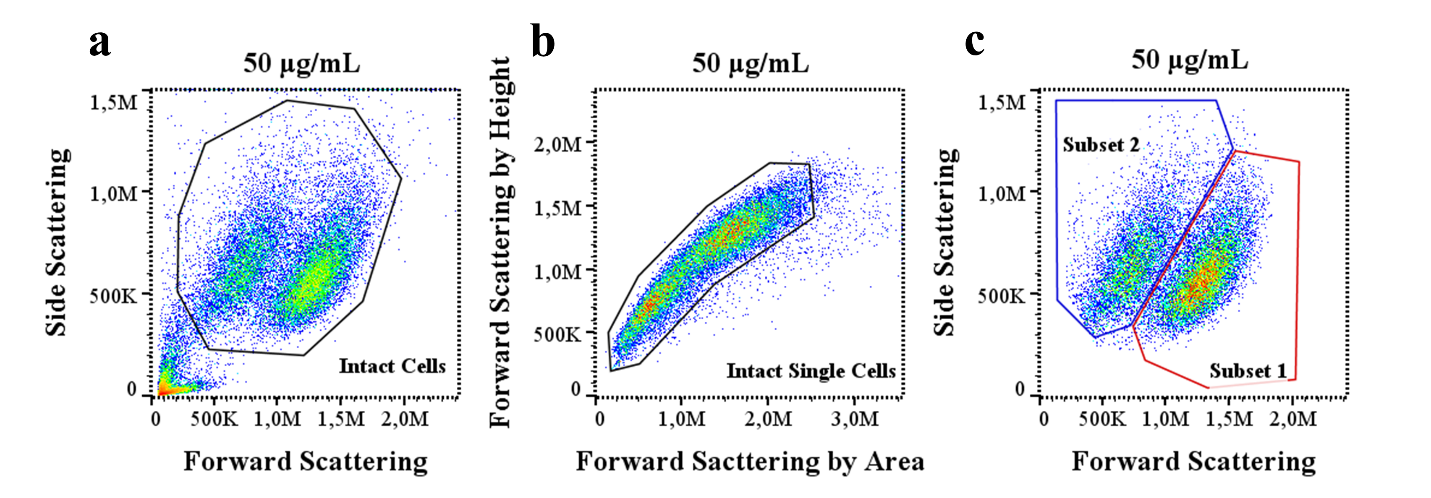


**Supplementary Fig. 3.** **Flow cytometry gating strategy for analysis of HeLa cells following incubation with LNPs.** Cells incubated with LNPs (50 µg/mL total lipids) for 4 h were first gated based on: (a) FSC vs SSC to exclude debris and select the intact cells, then (b) FSC-A vs FSC-H to exclude cell doublets and ensure analysis of single cells, and lastly (c) gates were set in the FSC vs SSC to separate the two cell subpopulations observed upon incubation with LNPs (hereafter referred to as subset 1 for the main cell sub-population, and subset 2 for the 2^nd^ subpopulation appearing upon incubation with LNPs).


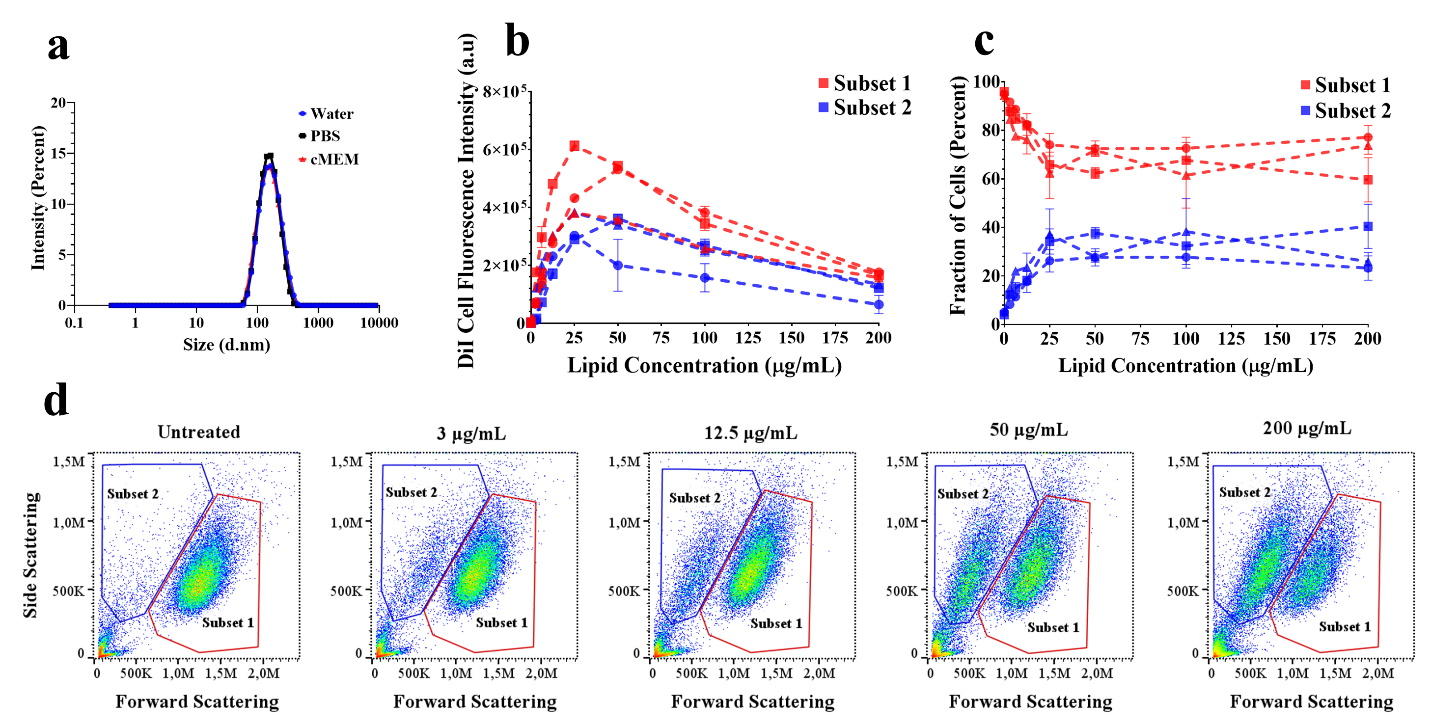


Supplementary Fig. 4. Uptake of LNPs prepared by vortexing by HeLa cells. (a) DLS size distributions of LNPs (100 µg/mL total lipids) prepared by vortex-mixing in different media (water, PBS and cMEM). (b) Median fluorescence intensity values obtained by flow cytometry of cells treated for 4 h with increasing concentrations of LNPs carrying poly-A and prepared by vortex-mixing. Red and blue symbols show the results of cells belonging to sub-population 1 and 2, respectively, as defined in the gates shown in panel d. (c) Percentage of cells in each sub-population. (d) Double scatter plots of forward versus side scattering of untreated HeLa cells as well as cells incubated with increasing concentrations of LNPs. The average and standard deviation of 3 replicate samples in 2 independent experiments are shown.


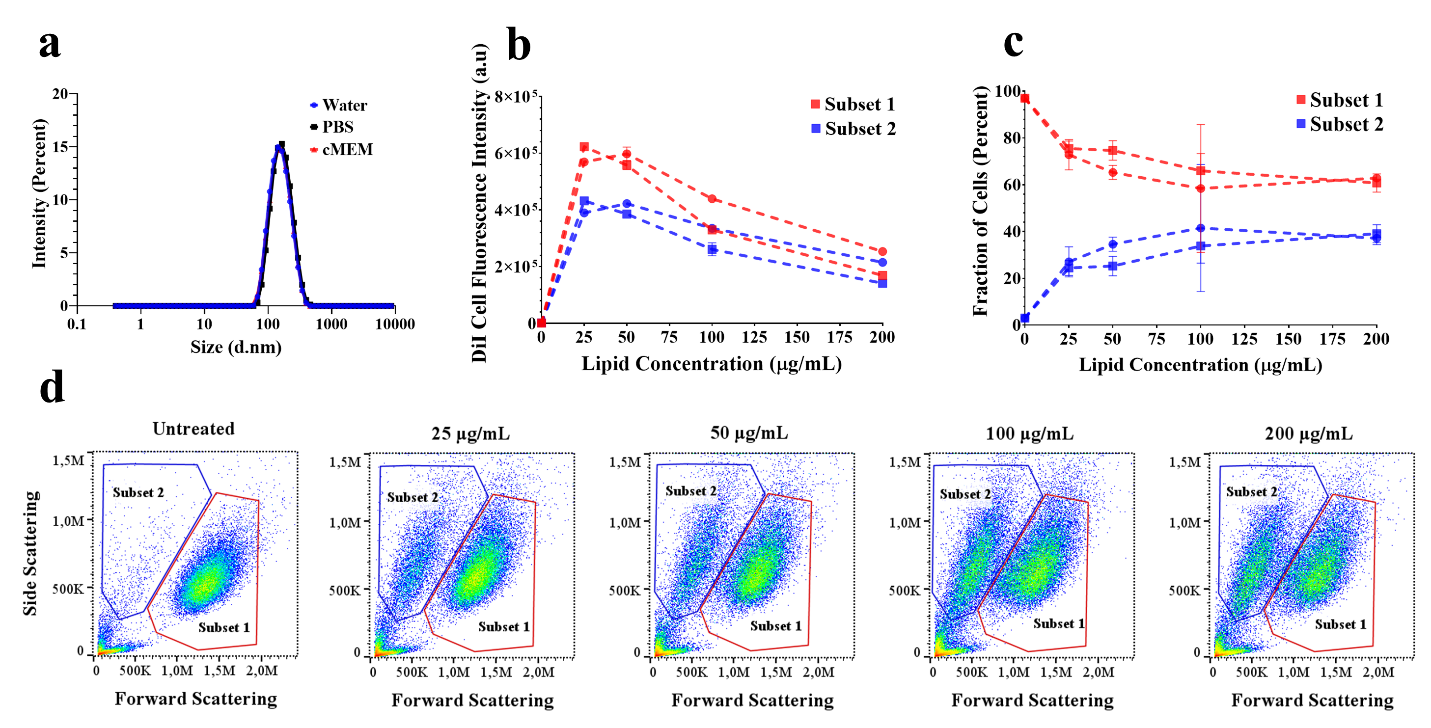


Supplementary Fig. 5. Uptake of empty LNPs by HeLa cells. (a) DLS size distributions of empty LNPs (not containing RNA) at 100 µg/mL total lipid concentration in different media (water, PBS and cMEM). (b) Median fluorescence intensity values obtained by flow cytometry of cells treated for 4 h with increasing concentrations of empty LNPs prepared by vortex-mixing. Red and blue symbols show the results of cells belonging to sub-population 1 and 2, respectively, as defined in the gates shown in panel d. (c) Percentage of cells in each sub-population. (d) Double scatter plots of forward versus side scattering of untreated HeLa cells as well as cells incubated with increasing concentrations of LNPs. The average and standard deviation of 3 replicate samples in 2 independent experiments are shown.


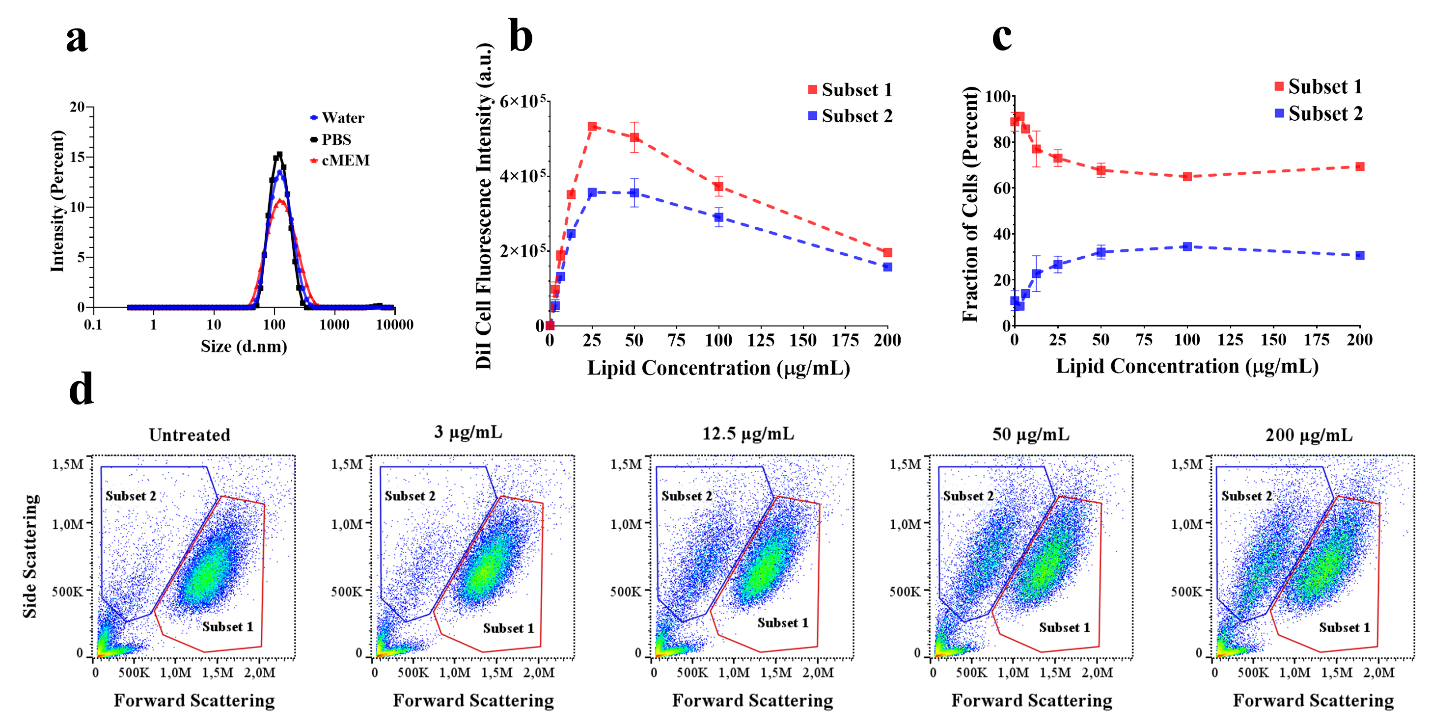


Supplementary Fig. 6. Uptake of LNPs at higher RNA content by HeLa cells. (a) DLS size distributions of LNPs at higher RNA content in different media (100 µg/mL total lipids in water, PBS and cMEM). (b) Median fluorescence intensity values obtained by flow cytometry of cells treated for 4 h with increasing concentrations of LNPs carrying poly-A in 10:1 w/w lipid to poly-A ratio. Red and blue symbols show the results of cells belonging to sub-population 1 and 2, respectively, as defined in the gates shown in panel d. (c) Percentage of cells in each sub-population. (d) Double scatter plots of forward versus side scattering of untreated HeLa cells as well as cells incubated with increasing concentrations of LNPs. The average and standard deviation of 3 replicate samples in 1 experiment are shown.


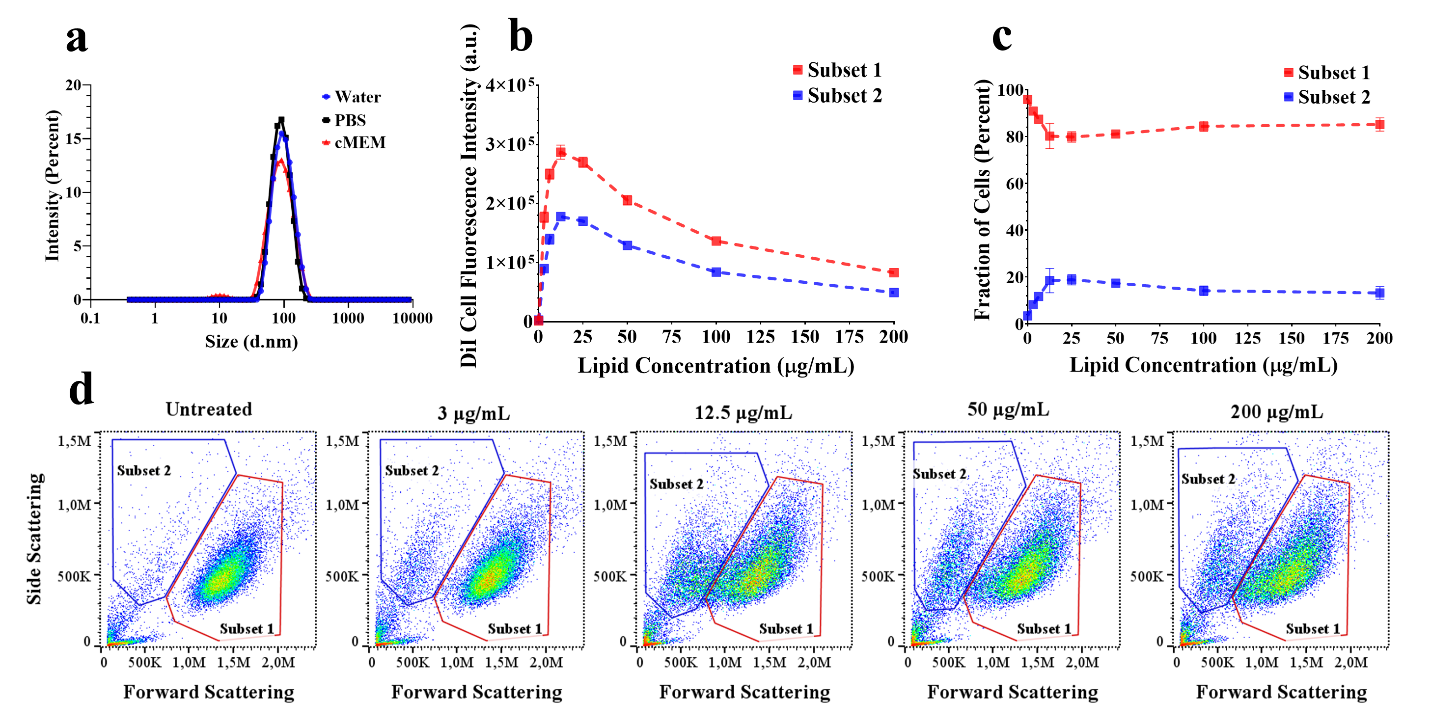


**Supplementary Fig. 7. Uptake of SM-102 LNPs at different concentrations in HeLa cells for 4 hours.** (a) DLS size distributions of LNPs in different media (50 µg/mL total lipids in water, PBS and cMEM). (b) Median fluorescence intensity values obtained by flow cytometry of cells treated for 4 h with increasing concentrations of LNPs carrying poly-A in 40:1 w/w lipid to poly- A ratio. Red and blue symbols show the results of cells belonging to sub-population 1 and 2, respectively, as defined in the gates shown in panel d. (c) Percentage of cells in each sub-population. (d) Double scatter plots of forward versus side scattering of untreated HeLa cells as well as cells incubated with increasing concentrations of LNPs. The average and standard deviation of 3 replicate samples in 1 experiment are shown.


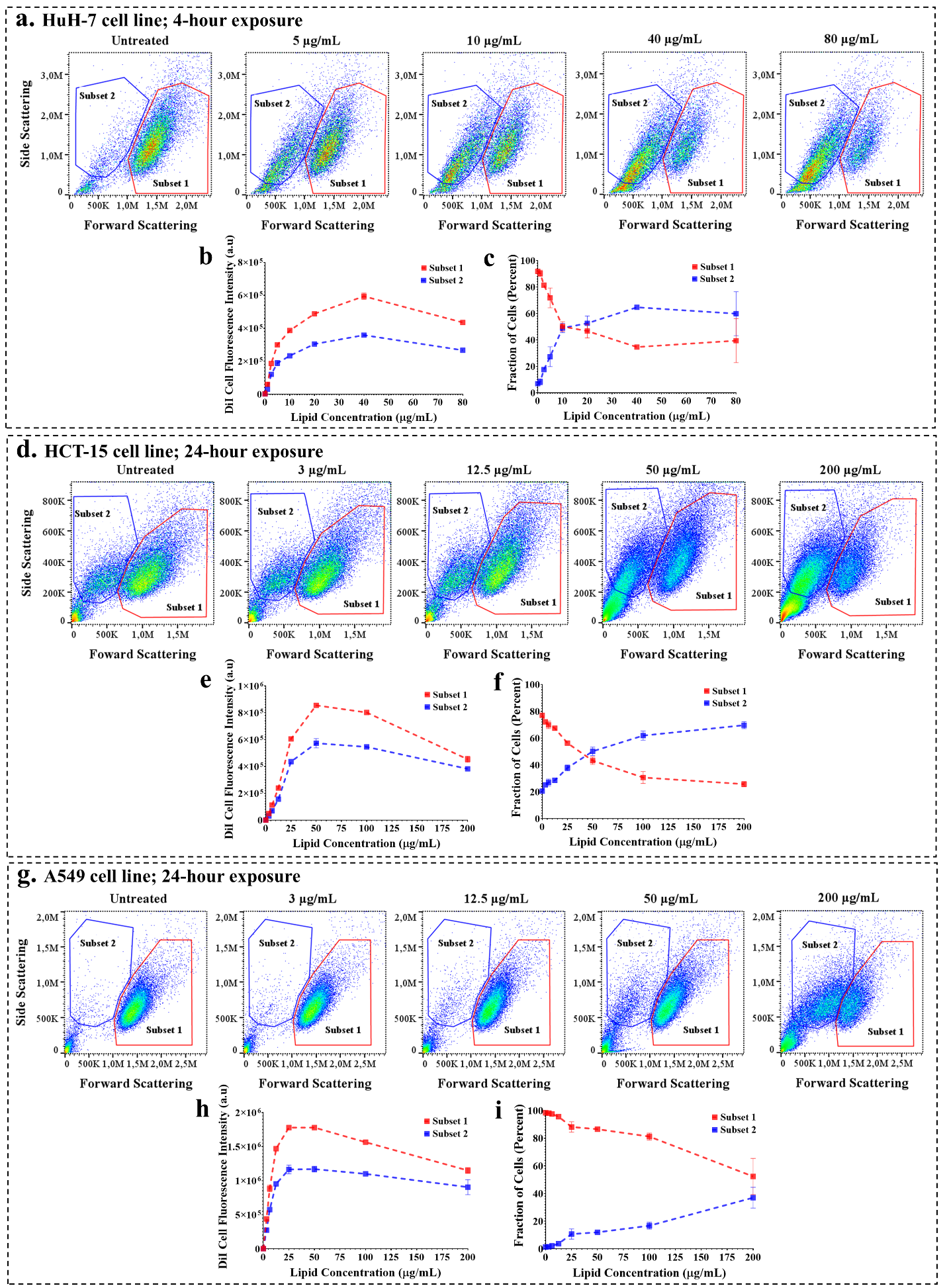


**Supplementary Fig. 8. Uptake of LNPs at different concentrations in various cell lines.** (a, d, g) Double scatter plots of forward versus side scattering of (a) HuH-7, (d) HCT-15, and (g) A549 cell lines incubated with increasing concentrations of LNPs. (b, e, h) Median fluorescence intensity values obtained by flow cytometry of (b) HuH-7, (e) HCT-15, and (h) A549 cell treated with increasing concentrations of LNPs. Red and blue symbols show the results for cells in subset 1 and 2, respectively, as defined in the gates shown in panel a, d, and g. (c, f, i) Percentage of cells in each sub-population. The results are the average and standard deviation of 2 replicate samples in one experiment.


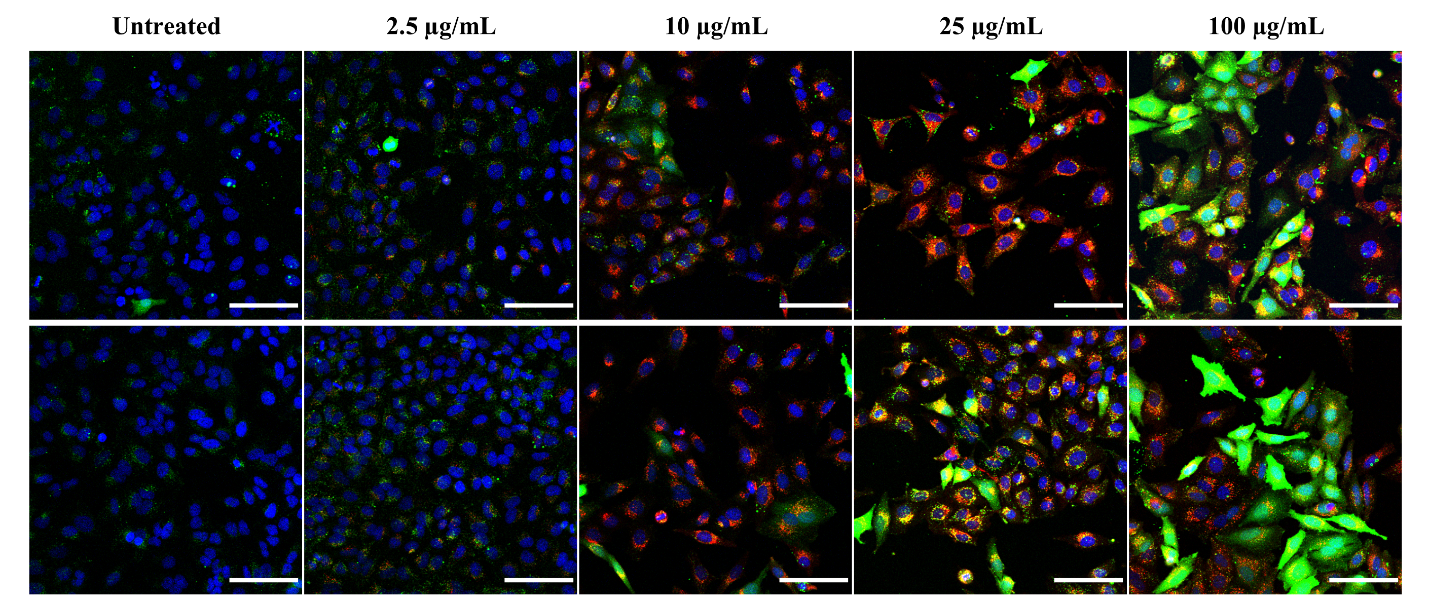


Supplementary Fig 9. Endosomal leakiness induced by LNPs. HeLa cells were treated with increasing doses of poly-A LNPs for 24 h followed by incubation with 3 mM calcein. Two images from replicate samples are shown for each condition.


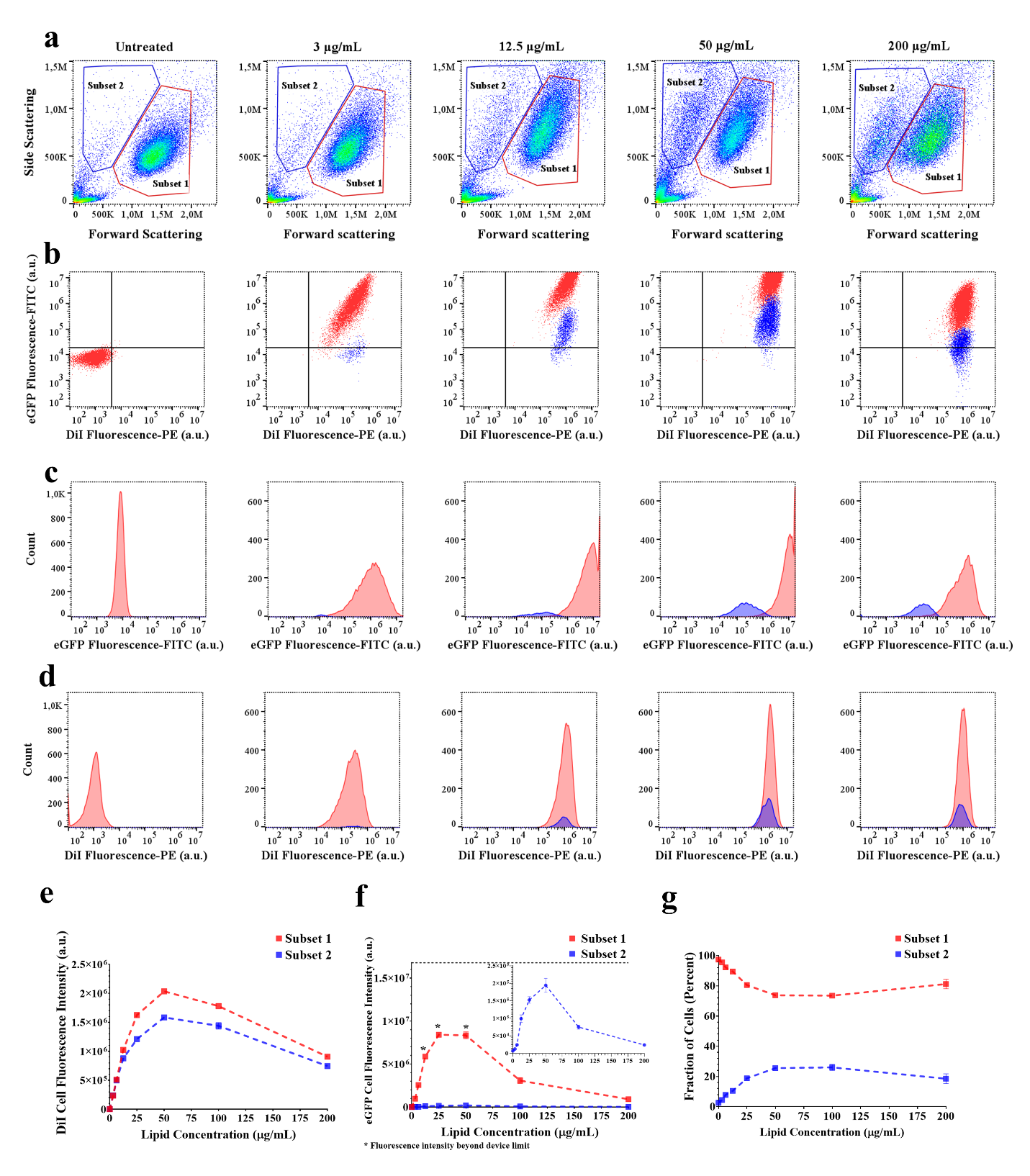


**Supplementary Fig. 10.** **Uptake and transfection of LNPs carrying the mRNA for GFP in HeLa cells after 24 h incubation.** (a) Forward-side scattering plots and (b) double scatter plots of transfection (eGFP fluorescence) versus uptake (DiI fluorescence) of untreated HeLa cells, as well as cells incubated with increasing concentrations of LNPs. (c-d) Fluorescence distributions of uptake (c) and transfection (d) at increasing concentrations of LNPs. Median fluorescence intensity values of (e) uptake and (f) transfection in cells treated with increasing concentration of LNPs for 24 h. Red and blue symbols show the results of cells belonging to sub-population 1 and 2, respectively, as defined in the gates shown in panel a. (g) Percentage of cells in each sub-population. The results are the average and standard deviation of 2 replicate samples in 1 experiment.


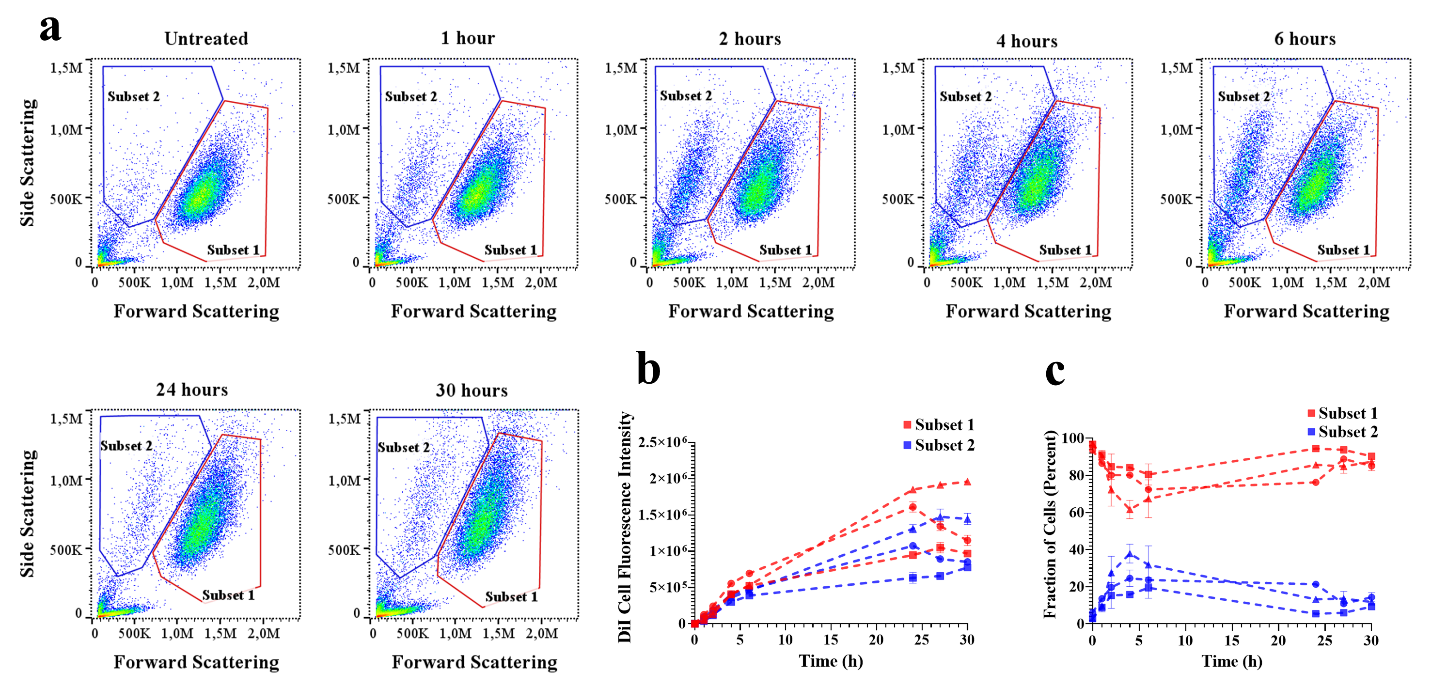


Supplementary Fig. 11. LNP uptake kinetics in HeLa cells. (a) Forward-side scattering plots of cells incubated with LNPs (12.5 µg/mL total lipids) carrying poly-A for increasing time. (b) Median cell fluorescence intensities over time. Red and blue symbols show the results of cells belonging to sub-population 1 and 2, respectively, as defined in the gates shown in panel a. (c) Percentage of cells in each sub-population. The average and standard deviation of 2 replicate samples in 3 independent experiments are shown.


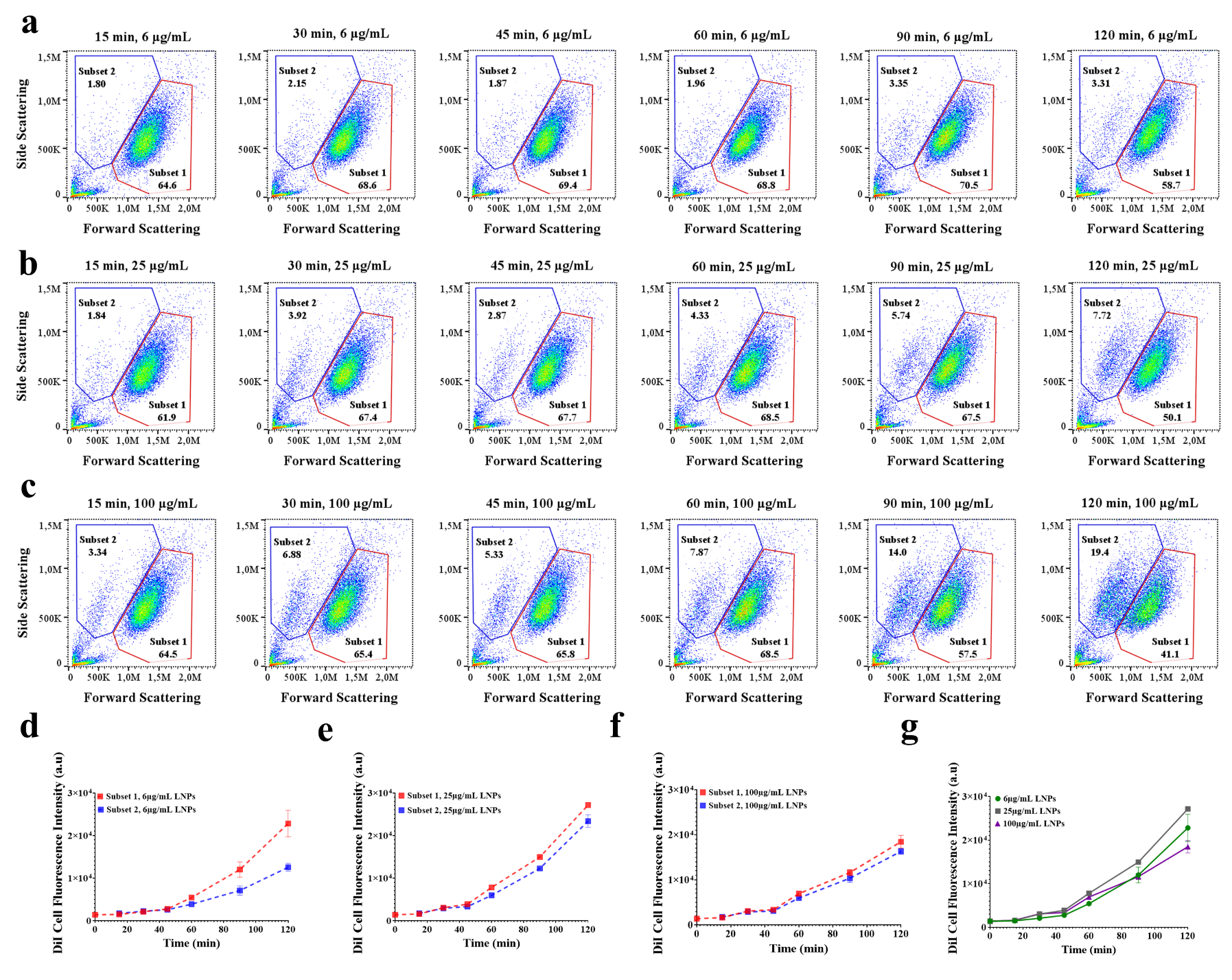


Supplementary Fig. 12. LNP uptake kinetics in HeLa cells. Forward-side scattering plots of cells incubated with poly-A LNPs at (a) 6 µg/mL, (b) 25 µg/mL, and (c) 100 µg/mL total lipid concentration for increasing time. Median cell fluorescence intensities of cell incubated with poly-A LNPs at (d) 6 µg/mL, (e) 25 µg/mL, and (f) 100 µg/mL total lipids as well as (g) an overlay of the uptake kinetics at the three concentrations. The average and standard deviation over two replicate samples of the results obtained in one experiment are shown.


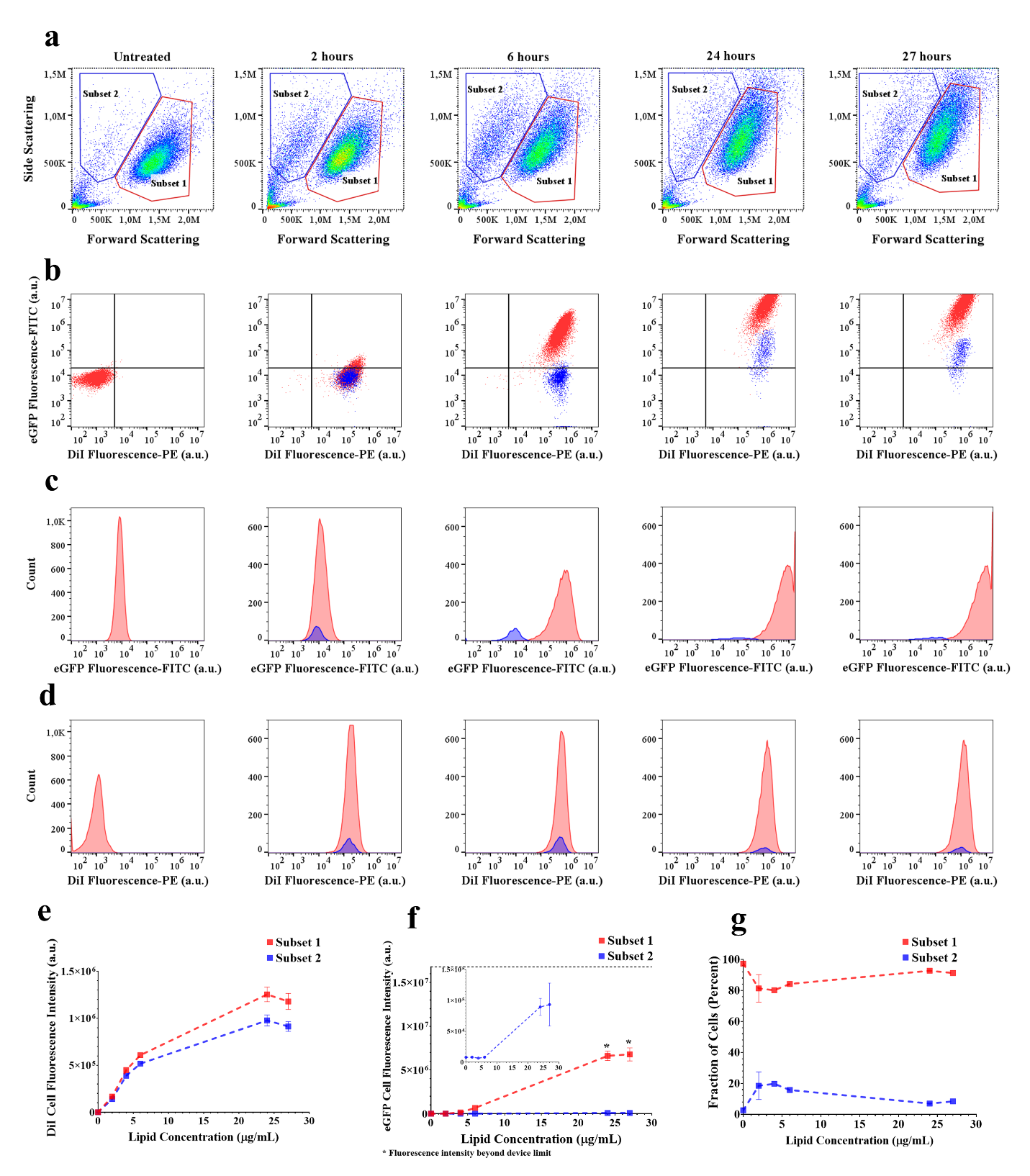


Supplementary Fig. 13. Uptake and transfection kinetics of LNP carrying the mRNA for GFP in HeLa cells. (a) Forward-side scattering plots and (b) double scatter plots of transfection (eGFP fluorescence) versus uptake (DiI fluorescence) of cells incubated with LNPs (12.5 µg/mL total lipids) for increasing time. Red and blue symbols show the results of cells belonging to sub-population 1 and 2, respectively, as defined in the gates shown in panel a. (c-d) Fluorescence distributions of uptake (c) and transfection (d) at different incubation times (subset 1 in red and subset 2 in blue). (e-f) Median fluorescence intensity values of (e) uptake and (f) transfection as a function of time. (g) Percentage of cells in each sub-population. The average and standard deviation of 2 replicate samples in 1 experiment are shown.

**
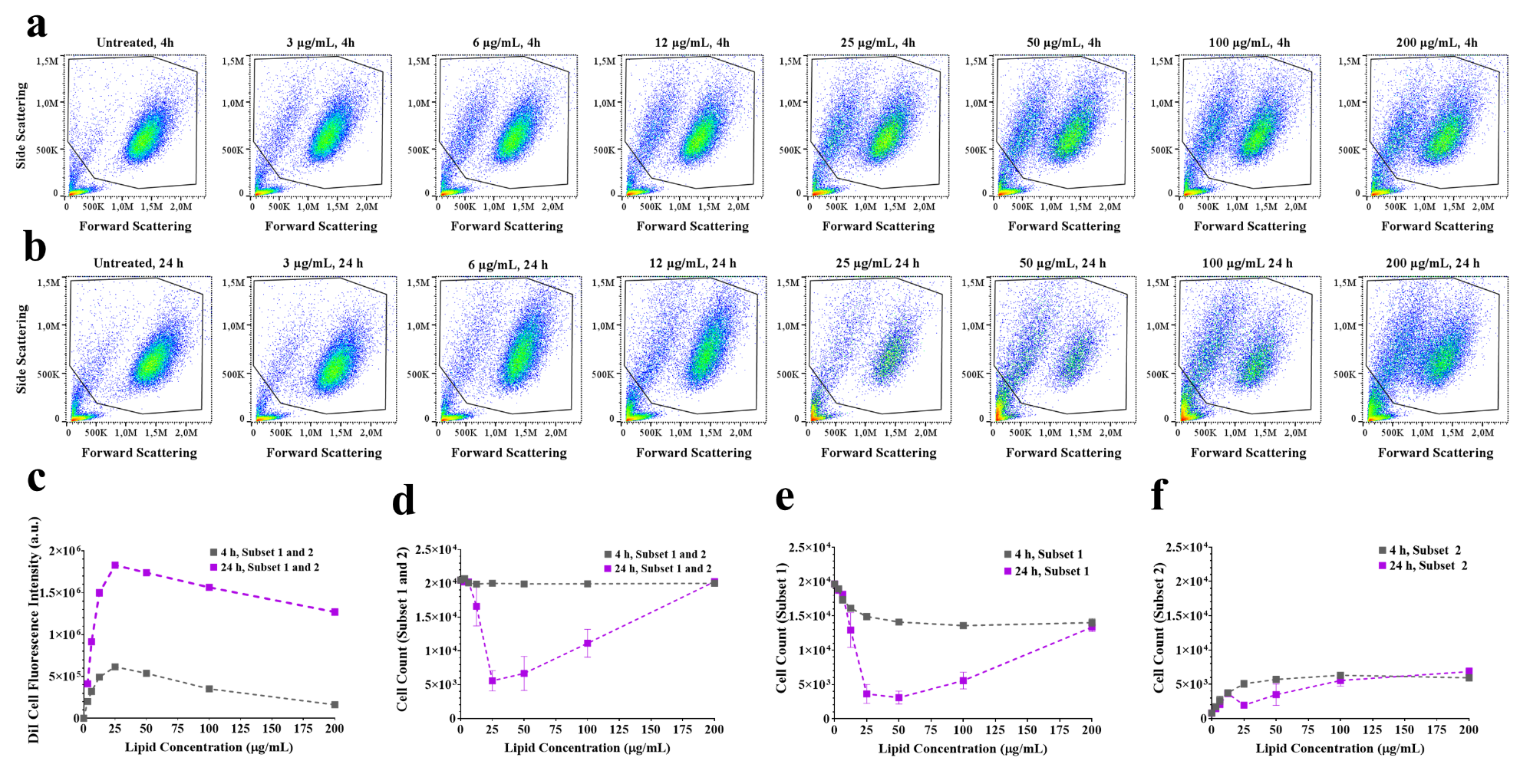
**

Supplementary Fig. 14. Uptake of LNPs at different concentrations in HeLa cells for 4 and 24 hours. Double scatter plots of forward versus side scattering of untreated HeLa cells as well as cells incubated with increasing concentrations of LNPs after (a) 4 and (b) 24 h incubation. (c) Median fluorescence intensity of all cells (including both subset 1 and 2 cells), after incubation with increasing concentrations of LNPs for 4 h (grey line) and 24 h (purple line). (d) Cell count (subset 1 and 2) after 4 and 24 h incubation with increasing concentrations of LNPs. (e) Subset 1 cell count after 4 and 24 h incubation with increasing concentrations of LNPs. (f) Subset 2 cell count after 4 and 24 h incubation with increasing concentrations of LNPs. The flow cytometer was set to acquire 20,000 cells for each sample, however in some samples a lower cell count was acquired, suggesting cell loss upon incubation with the LNPs. The average and standard deviation over two replicate samples of the results obtained in one experiment are shown.


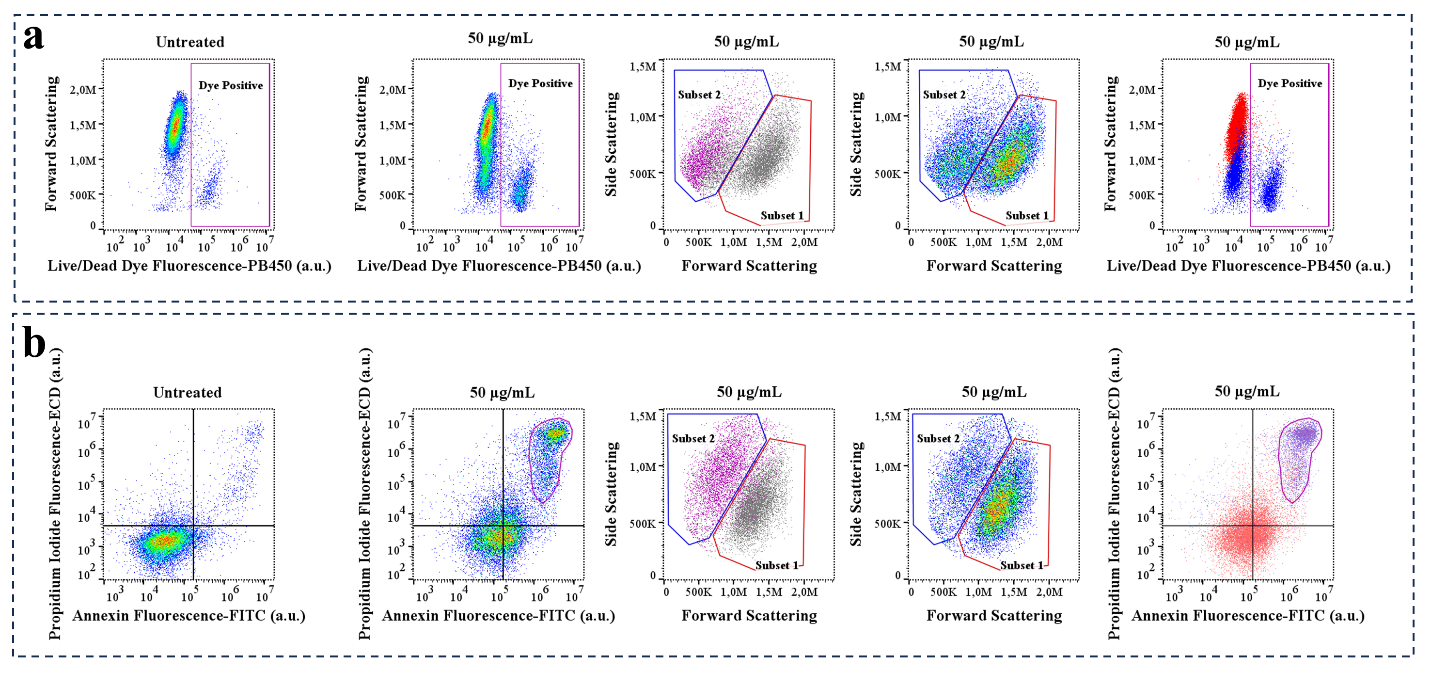


**Supplementary Fig. 15. Flow cytometry gating strategy for analysis of HeLa cells following LNP exposure and viability staining:** (a) live/dead fixable viability dye eFluorTM 450, as well as (b) propidium iodide/ Annexin V staining*.* After excluding debris and doublets and setting gates to identify subset 1 and subset 2 cells as shown in Supplementary Fig.3, untreated cells (stained with the viability dyes) and LNP treated cells were gated to set thresholds for dye positivity using dye fluorescence channels. Next, the dye-positive gate is applied in the FSC vs SSC double scatter plots in order to determine whether the dye-positive cells belong to specific subpopulations (subset 1 and subset 2). Similarly, the gates of subset 1 and subset 2 cells are applied in the viability dye scatter plots in order to determine their distribution among the dye positive and dye negative cells.


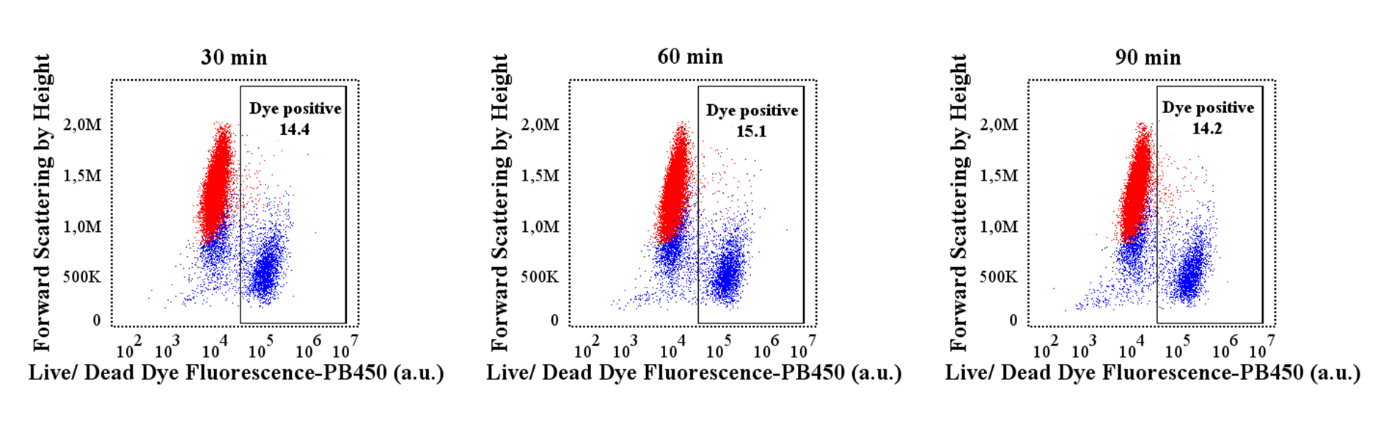


Supplementary Fig. 16. Optimization of staining conditions for Live/dead fixable viability dye eFluor^TM^ 450 for HeLa cells. Double scatter plots of forward scattering versus Live/dead fixable viability dye eFluor^TM^ 450 intensity of HeLa cells incubated with LNPs (50 µg/mL total lipids) for 4 h and then stained with the dye for different times (30, 60 and 90 min staining). The average and standard deviation over 3 replicate samples of the results obtained in one experiment are shown. The percentage of dye positive cells was similar (14-15%) even when increasing the staining time.


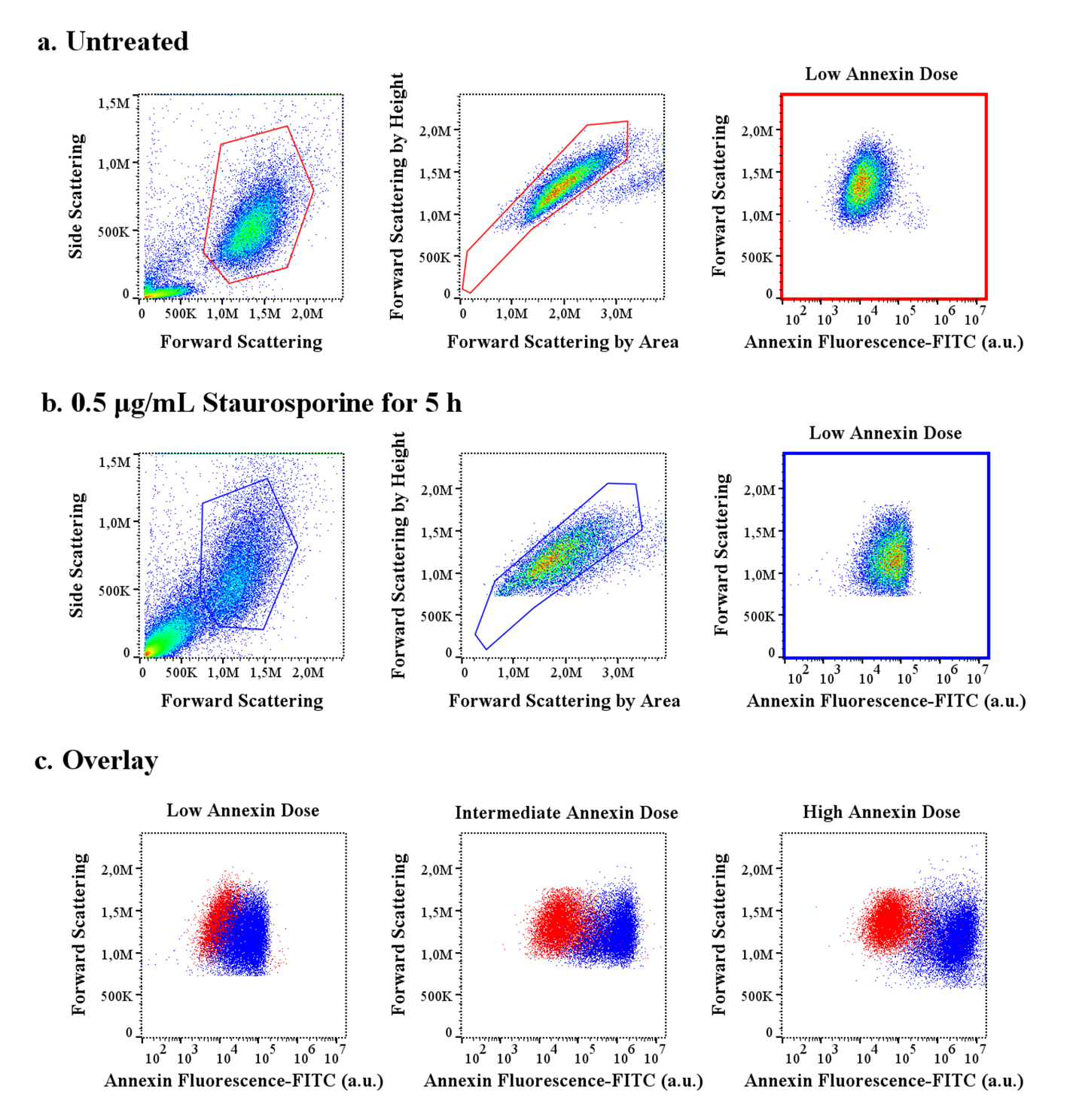


Supplementary Fig. 17. Optimization of staining conditions for FITC-Annexin V viability dye for HeLa cells. Flow cytometry double scatter plots of (a) untreated cells (labelled in red), and (b) apoptotic cells (HeLa cells treated with 0.5 µg/mL staurosporine for 5 h, labelled in blue) after incubation with low, intermediate and high FITC-Annexin V dye amounts (2000, 200 and 20 times dilution respectively). In (c) the same results are overlapped in the same plots, confirming that the protocol allowed to distinguish healthy and apoptotic cells. Based on these results, a 200-fold dilution was used for further experiments. The results obtained in one experiment with 3 replicate samples are shown.


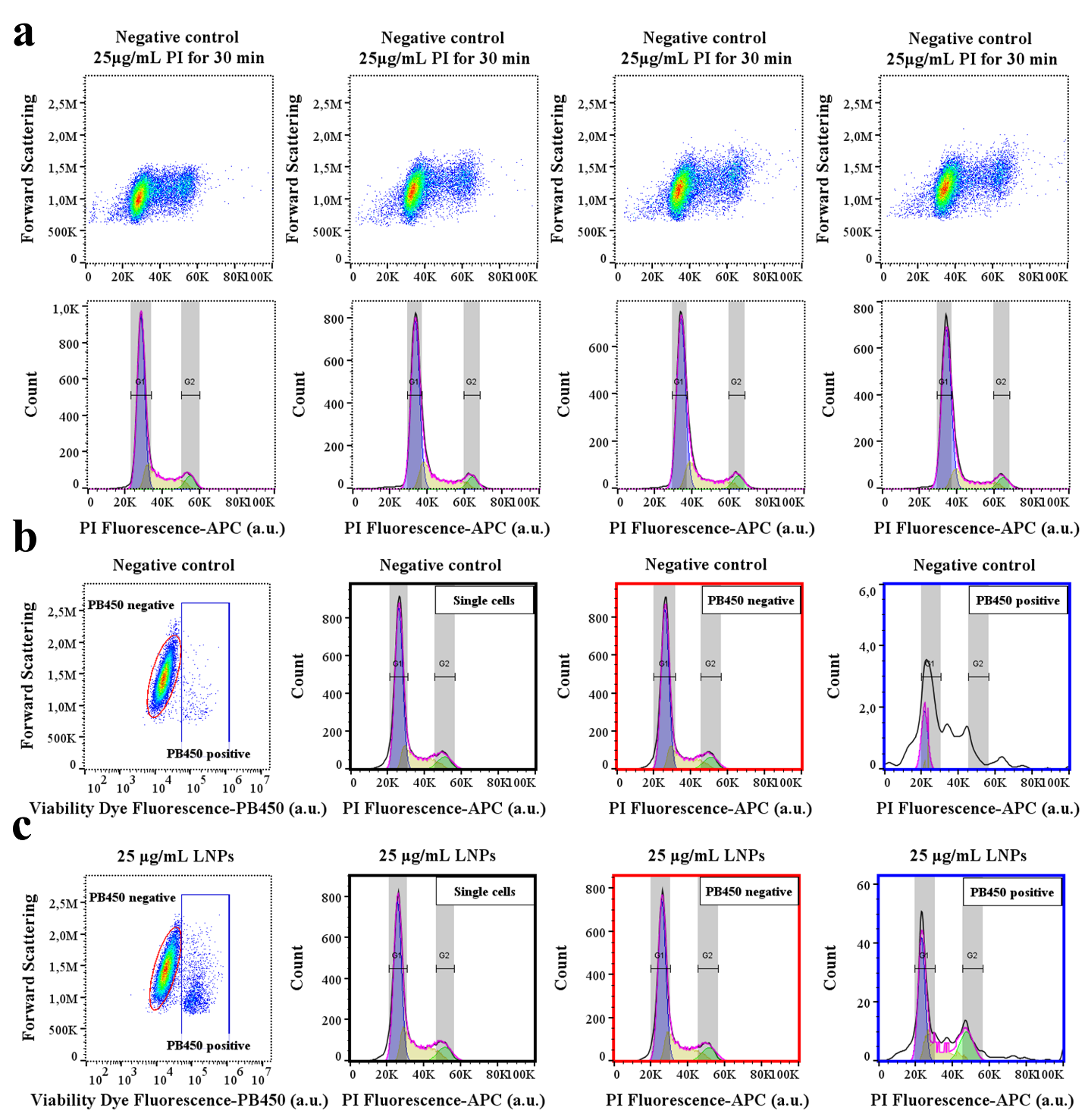


Supplementary Fig. 18. Cell cycle distribution of HeLa cells incubated with LNPs. (a) Double scatter plots of HeLa cells incubated with PI at different concentrations (25 and 50 µg/mL) for different incubation times (30, 75 and 120 min). (b-c) Double scatter plots and PI distribution of (b) untreated HeLa cells and (c) HeLa cells incubated with DiI-free LNPs (25 µg/mL total lipids). Cells were fixed and permeabilized as described in the Methods and then stained with the live/dead fixable viability dye eFluor^TM^ 450 and PI (total DNA staining) in order to label subset 2 cells and determine their cell cycle distribution. The results obtained in one experiment with 3 replicate samples are shown. No major differences in the cell cycle distribution could be seen between the viable cells (subset 1) and cells of subset 2 labelled with the viability dye.


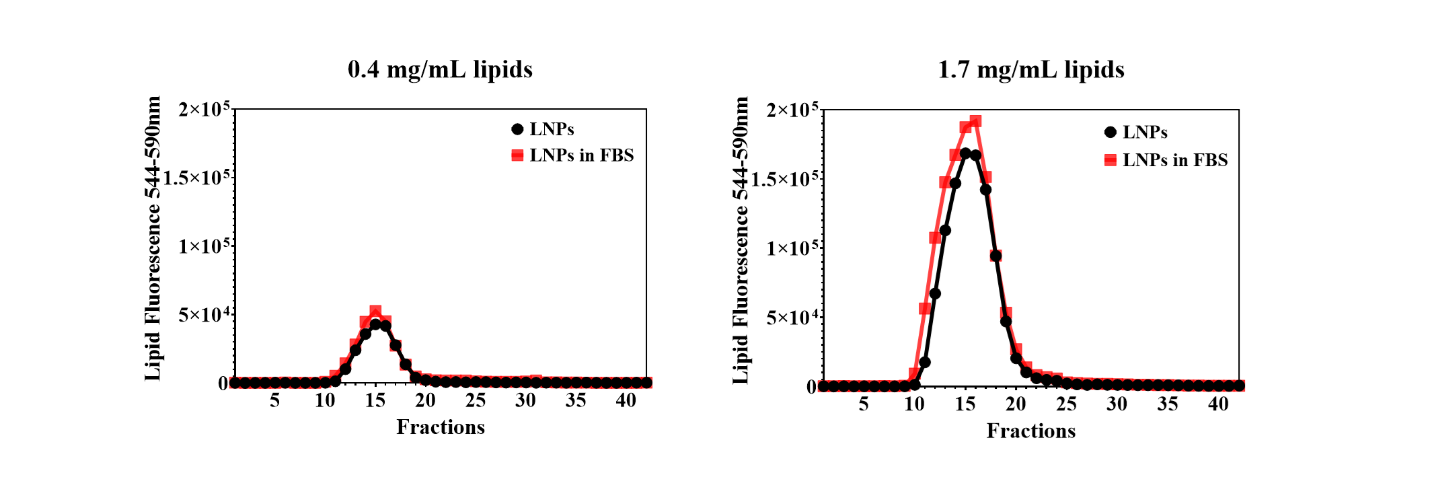


Supplementary Fig. 19. Comparative elution profiles of LNP in buffer and in FBS. Elution profiles by SEC of LNPs at two different total lipid concentrations in buffer and in 77% FBS (~31 mg/mL proteins). The elution profiles were obtained by measuring the fluorescence of DiI in the different fractions (Excitation-emission 544-590 nm). The results show that the early peak where LNPs elute has the same intensity for LNP in buffer and LNP at higher serum concentration, hence suggesting that transfer of DiI to serum components is not observed. Similarly, no DiI fluorescence is detected in all later fractions where the free serum proteins elute, again suggesting lack of transfer of DiI to serum components.


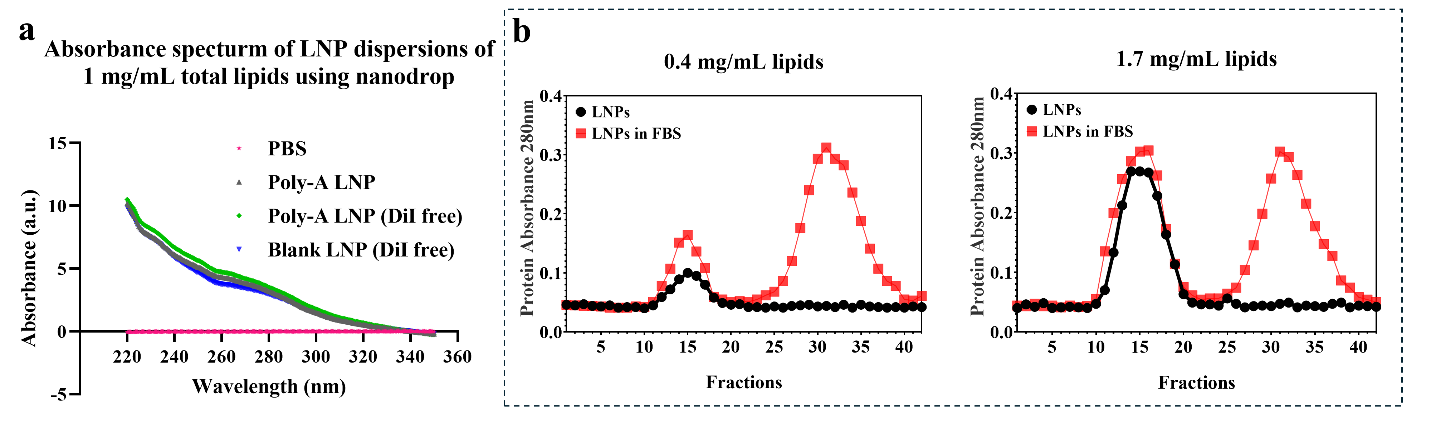


**Supplementary Fig. 20.** UV-visible absorbance of different LNP formulations. (a) Absorbance spectra of LNP dispersions. (b) Elution profiles obtained by SEC of LNPs at two different total lipid concentrations in buffer or in a dispersion with 77% FBS (~ 31 mg/mL proteins). The results show that absorbance at 280 nm is observed also for LNP in buffer without proteins added (a). Consecutively the SEC elution profile at 280 nm show a peak in the early fractions where LNP elute even for samples of LNP in buffer without proteins. Because of this, the LNP elution peak could not be used for corona protein quantification.
